# Chemical synthesis of a reported p47phox/p22phox inhibitor and characterization of its instability and irreproducible activity

**DOI:** 10.3389/fphar.2022.1075328

**Published:** 2023-01-05

**Authors:** Jie Zang, Yves Cambet, Vincent Jaquet, Anders Bach

**Affiliations:** ^1^ Department of Drug Design and Pharmacology, Faculty of Health and Medical Sciences, University of Copenhagen, Copenhagen, Denmark; ^2^ READS Unit, Centre Médical Universitaire, University of Geneva, Geneva, Switzerland; ^3^ Department of Pathology and Immunology, Centre Médical Universitaire, University of Geneva, Geneva, Switzerland

**Keywords:** NADPH (nicotinamide adenine dinucleotide phosphate) oxidase, NOX2, p47phox, LMH001, oxidative stress, reactive oxygen species, protein-protein interactions (PPIs), chemical probes

## Abstract

The nicotinamide adenine dinucleotide phosphate oxidase 2 (NOX2) multi-subunit complex is a highly abundant and central source of reactive oxygen species. NOX2 is a key enzyme of the innate immune system involved in antibacterial response, but excessive NOX2 activity is involved in oxidative stress and inflammation in many diseases. Inhibition of NOX2 has great potential as a therapeutic strategy. An intriguing pharmacological approach for inhibiting NOX2 is to target the p47phox subunit and thereby block the protein-protein interaction with p22phox, whereby assembling and activation of NOX2 is prevented. However, the shallow binding pocket of p47phox makes it difficult to develop drug-like p47phox/p22phox inhibitors. Recently, the small molecule LMH001 was reported to inhibit the p47phox/p22phox interaction, reduce endothelial NOX2 activity, and protect mice from angiotensin II-induced vascular oxidative stress. These noteworthy results could have significant impact on the field of NOX2 pharmacology, as specific and efficient inhibitors are scarce. Here, we synthesized and tested LMH001 to have it available as a positive control. We established a robust synthetic route for providing LMH001, but subsequently we experienced that LMH001 is chemically unstable in aqueous buffer. In addition, neither LMH001 nor its breakdown products were able to inhibit the p47phox/p22phox interaction in a non-cellular fluorescence polarization assay. However, LHM001 was a weak inhibitor of NOX2 in a functional cell assay, but with same low potency as one of its breakdown products. These findings question the activity and suggested mechanism of LMH001 and constitute important information for other researchers interested in chemical probes for studying NOX2 biology.

## Introduction

**FIGURE 1:**
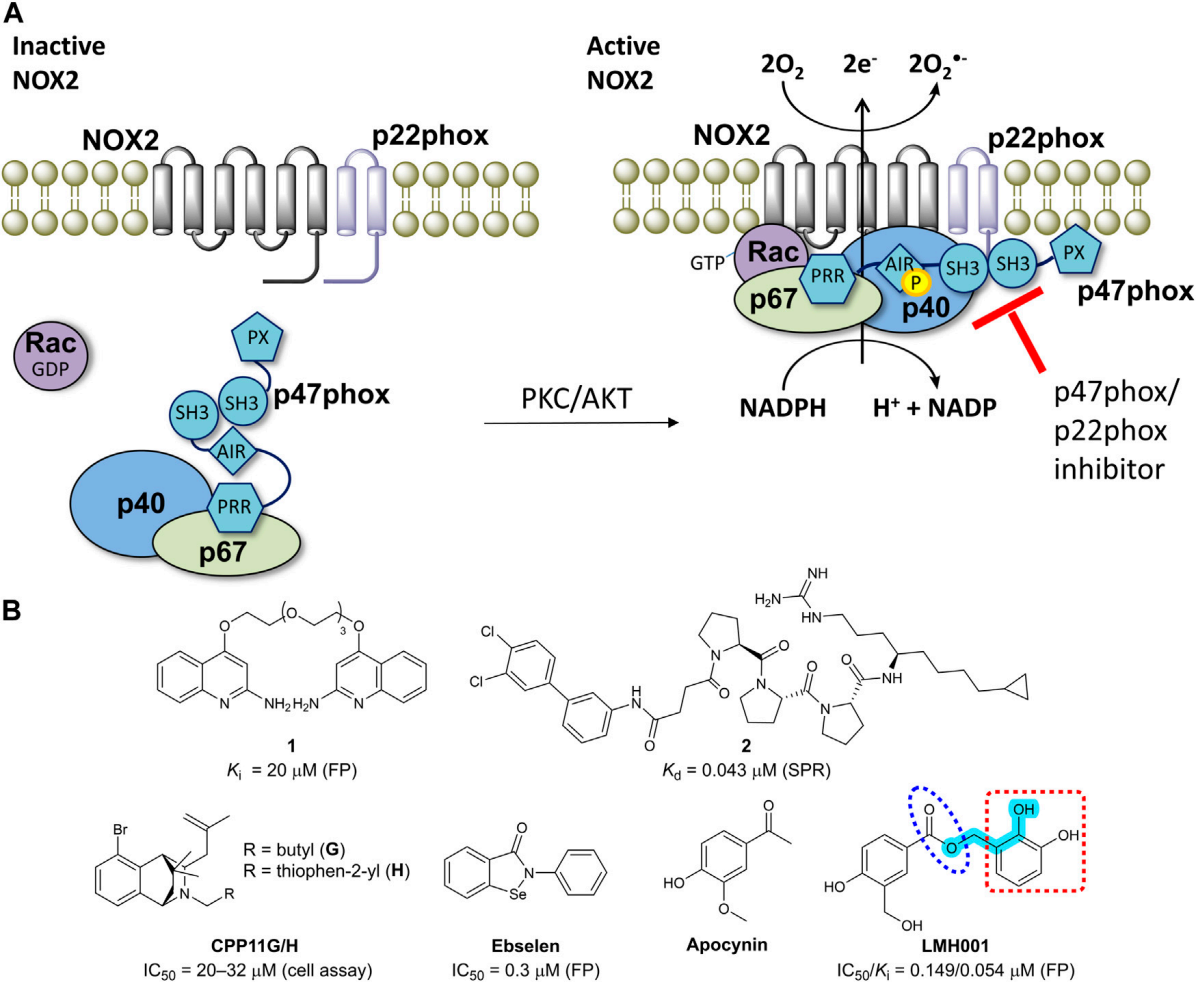
**(A)** The mechanism of NOX2 activation. At resting state, the cytosolic and membrane-bound subunits are separated. When p47phox is activated by the kinases (protein kinase C, PKC or protein kinase B, also known as AKT), the cytosolic subunits translocate to the membrane and assemble with the membrane-bound subunits to form an active NOX2 complex. **(B)** Chemical structures of reported small-molecule p47phox/p22phox inhibitors. For LMH001, key parts are marked by turquoise color (Mannich base-like group), blue circle (ester), and a red square (catechol moiety).

NADPH oxidase 2 (NOX2) is a multi-subunit enzyme complex and a main source of reactive oxygen species (ROS). In phagocytes, NOX2 produces ROS in relation to respiration burst as a defense mechanism against bacterial infections (Bedard and Krause, 2007). In other cell types including endothelial cells, B lymphocytes, and microglia, NOX2 generates superoxide as part of physiologically relevant redox signaling events. However, excessive and pathological ROS production by NOX2 contributes to oxidative stress and inflammation in connection to a wide range of diseases (Drummond et al., 2011; Diebold et al., 2015; Casas et al., 2020). NOX2 is activated by phosphorylation of p47phox, leading to translocation of p47phox and p67phox to the membrane and thus assembling and activation of the entire NOX2 complex (Figure 1A) (Bedard and Krause, 2007; Drummond et al., 2011).

Due to its central role in disease, NOX2 is an attractive drug target. However, developing high-quality chemical probes (Arrowsmith et al., 2015; Blagg and Workman, 2017) and preclinical drugs for NADPH oxidases has proven very difficult. Current NOX inhibitors mostly bind the transmembrane catalytic core subunit and suffer from off-target binding, lack of selectivity among the isoforms (NOX1-5 and DUOX1-2), and intrinsic anti/pro-oxidative effects, which complicate the interpretation of biological results (Wind et al., 2010; Altenhofer et al., 2015). Recently, two studies established that the apparent activity of most known NOX inhibitors was due to chemical liabilities leading to assay-interference and off-target effects (Augsburger et al., 2019; Reis et al., 2020). Overall, the field of NOX drug discovery is in great need of true and reliable inhibitors with the specificity, selectivity, and pharmacokinetic properties suitable for biological target validation.

One way of reducing NOX2 activity is to target the intracellular organizer subunit p47phox with small molecules. This approach inhibits the protein-protein interaction (PPI) between p47phox and p22phox (Figure 1A), thereby preventing assembling and activation of the NOX2 complex leading to diminished levels of superoxide (Drummond et al., 2011; Diebold et al., 2015). This strategy has potential to provide isoform selective NOX inhibitors, as p47phox is essential for NOX2 activity, but not involved in the activation of the other NADPH oxidases (except, partly, for NOX1 in smooth muscle cells). However, the SH3A−B domain of p47phox that accommodate the proline-rich region (PRR) of p22phox makes a shallow binding pocket, for which discovery of high-affinity small-molecules is challenging (Drummond et al., 2011).

Several p22phox mimicking peptides (Leto et al., 1994; Shi et al., 1996; Dahan et al., 2002; Drummond et al., 2011) and a few small-molecules have been reported as p47phox/p22phox inhibitors (Figure 1B). Recently, the polyethylene glycol (PEG)-based dimeric compound **1** (Solbak et al., 2020) and the peptide-derived triproline mimetic **2** (Garsi et al., 2022) were identified by fragment-based drug discovery and structure-based design, respectively. Both were shown to directly bind the SH3A−B domain of p47phox by surface plasmon resonance (SPR), and **1** inhibits the binding between a fluorescently-labelled p22phox peptide and p47phox^SH3A−B^ in a cell-free fluorescence polarization (FP) assay. However, despite binding specifically to the target, p47phox, cell-based activity data are lacking for both **1** and **2**. In contrast, CPP11G and CPP11H block translocation of p47phox to the membrane and diminish the subsequent NOX2 assembling and activity in cells (Li et al., 2019). Molecular docking led to the hypothesis that CPP11G and H bind p47phox^SH3A−B^ and inhibit the interaction with p22phox or the catalytic NOX2 subunit, but this was not supported by biophysical evidence for a direct binding to p47phox or cell-free assay data showing p47phox/p22phox inhibition. Ebselen was shown to inhibit the p47phox/p22phox interaction in FP and block p47phox translocation to neutrophil membranes (Smith et al., 2012). However, ebselen is a problematic and unspecific molecule with various scavenging, assay-interference, and off-target properties (Altenhofer et al., 2015; Augsburger et al., 2019; Reis et al., 2020). In fact, ebselen aggregates p47phox *via* a covalent mechanism (Solbak et al., 2020).

Recently, LMH001 (Figure 1B) was presented as a new small-molecule p47phox/p22phox inhibitor able to reduce endothelial NOX2 activity and protect mice (but not p47phox knockout mice) from angiotensin II-induced vascular oxidative stress, hypertension and aortic aneurysm (Fan et al., 2022). LMH001 was computationally designed to target the p47phox/p22phox interface based on a crystal structure of p47phox^SH3A−B^ in complex with a short peptide derived from the p22phox PRR. Accordingly, LMH001 inhibited the interaction between glutathione S-transferase (GST)-tagged p47phox^SH3A−B^ and a fluorescently FITC-labelled p22phox peptide.

LMH001 could provide significant progress to the field, considering the lack of reliable and potent chemical probes for studying p47phox/p22phox inhibition. Thus, incited by the results in Fan et al., we synthesized and tested LMH001 with the aim of using it as a positive control in future studies.

## Material and methods

### Chemistry

LMH001 was obtained as white solid in high purity (>95%) by chemical synthesis in nine steps. The final product was thoroughly characterized by 1D NMR (^1^H NMR, ^13^C NMR), 2D NMR (^1^H–^1^H COSY, ^1^H-^13^C HSQC, ^1^H-^13^C HMBC), and liquid chromatography–mass spectrometry (LC−MS). The synthetic procedures are described in Supplementary Material.

### Fluorescence polarization (FP) assay

First, saturation experiments were performed to determine the binding affinities (*K*_d_ values) between the fluorescent peptide probes (cyanine 5 (Cy5)-p22phox^149−162^ and carboxytetramethylrhodamine (TAMRA)-p22phox^151−162^) and p47phox^SH3A−B^ (human His-tagged p47phox^151−286^; sequence in Supplementary Material) in 1×HBST buffer (50 mM Hepes, 150 mM NaCl and 0.005% Tween 20, pH 7.4), as previously described (Solbak et al., 2020). Increasing concentrations of p47phox^SH3A−B^ (two-fold dilution series, 12 points as duplicates, 0–50 μM for Cy5-p22phox^149−162^ and 0–131 µM for TAMRA-p22phox^151−162^) were added to a fixed concentration of the p22phox probe (5 nM Cy5-p22phox^149−162^; 10 nM TAMRA-p22phox^151−162^) in a final DMSO concentration of 2%. Assay plates were incubated in darkness for 2 min (explorative method) or 20 min (standard method) at room temperature before measuring the FP levels. The Cy5- and TAMRA-probes were measured at excitation/emission values of 635/670 and 530/585 nm, respectively. *K*_d_ values were derived by fitting the FP values to the one-site specific binding equation: Y = B_max_ × X/(*K*_d_ + X), with B_max_ being the maximal FP value, X is the p47phox^SH3A−B^ concentration, and Y is the variable FP values.

Synthesized compounds were then tested for their ability to inhibit the interaction between p47phox^SH3A−B^ and the fluorescent peptide probes in 1×HBST (2% DMSO). The non-fluorescent p22phox^151−162^ peptide (Sequence: Ac-PPTNPPPRPPAE-NH_2_) was used as a control. The assay was performed by adding increasing concentrations of the test compounds (2-fold dilution, 12 points as duplicates) to a fixed concentration of p47phox^SH3A−B^ (1.25 µM for Cy5-p22phox^149−162^; 4 µM for TAMRA-p22phox^151−162^) and peptide probes (5 nM Cy5-p22phox^149−162^; 10 nM TAMRA-p22phox^151−162^) using the same conditions as described above. FP values were fitted to the equation Y = Bottom + (Top-Bottom)/{1 + 10^[(LogIC_50_-X)*HillSlope]}, where X is the logarithmic value of the compound concentration. Hereby, the IC_50_ value was obtained, which together with the *K*_d_ value, probe and p47phox^SH3A−B^ concentrations was used to calculate the theoretical competitive inhibition constant, the *K*_i_ value. An additional explorative experiment of testing the activity of LMH001 was done in water instead of 1×HBST buffer. Here, 5 and 8 µM of p47phox^SH3A−B^ concentration was used for Cy5-p22phox^149−162^ and TAMRA-p22phox^151−162^, respectively.

### Chemical stability assay

The stability of LMH001 was tested by electron spray ionization (ESI) LC−MS using an Agilent 6130 mass spectrometer instrument coupled to an Agilent 1200 high-performance liquid chromatography system installed with a C18 reversed-phase column (Zorbax Eclipse XBD-C18, 4.6 mm × 50 mm) and a diode array detector. A binary solvent system of buffer A (H_2_O/acetonitrile (MeCN)/formic acid, 95:5:0.1 v/v%) to buffer B (MeCN/formic acid, 99.9:0.1 v/v%) and a flow rate of 1 mL/min was used. Injection volume was 5 µL. Total sample run time was 6 min, and the percentage of buffer B increases from 0% to 100% during 0.25–4.25 min, maintains at 100% until 5 min, then decreases to 0% at 5.10 min, and stays at 0% until 6 min. A 10 mM DMSO stock of LMH001 was diluted into 100 µM in different assay buffers including MeCN, water, 1 × PBS (137 mM NaCl, 2.7 mM KCl, 10 mM Na_2_HPO_4_, 1.8 mM KH_2_PO_4_, pH = 7.4), HBSS (Hanks’ Balanced Salt solution, ThermoFisher, Catalog number: 14025050), 100 mM PPB (61 mM K_2_HPO_4_, 39 mM KH_2_PO_4_, pH = 7.4), 1×HBST, Hepes (50 mM Hepes, pH = 7.4), Tris (10 mM Tris, pH = 7.4), and a 63.5:36.5:0.1 v/v% mix of H_2_O/MeCN/trifluoroacetic acid (TFA) and incubated for 5 and 30 min before analysis by LC−MS. The percent LMH001 remaining over time was calculated relative to 5 min of LMH001 in MeCN.

### Covalent binding assay

LMH001 was tested for covalent reactivity towards p47phox^SH3A−B^ by LC−MS, as described previously for ebselen (Solbak et al., 2020). 200 µM of LMH001 or ebselen was incubated at room temperature with p47phox^SH3A−B^ (0.2 mg/mL) in buffer (1×HBST) or water for 15 min before analysis by LC−MS. LC−MS spectra were obtained using the same LC–MS system, solvents, and gradient as for the chemical stability assay, but here the LC–MS was installed with a Poroshell C18 reverse-phase column (Agilent, Poroshell, 300SB-C18, 2.1 × 75 mm). Injection volume was 10 µL and the protein eluted at 3.2 min.

### NOX2 cell-based assay

NOX2 activity was measured as previously described with small modifications (Augsburger et al., 2019). The assay measures extracellular superoxide generated by PMA-activated NOX2 in PLB-985 cells using the cell-impermeable tetrazolium salt WST1 as probe (Tan and Berridge, 2000). PLB-985 cells (Tucker et al., 1987) were grown in suspension in 75 cm^2^ culture flasks in RPMI-1640 medium (GIBCO; #61870-010) containing 10% foetal bovine serum and penicillin/streptomycin (50 U/mL) at 5% CO_2_/95% air in a humidified atmosphere at 37°C. PLB-985 cells (2 × 10^5^ cells/mL) were differentiated into granulocyte-like cells by addition of DMSO (1.25% final concentration) for 4–5 days. DMSO stocks (100 mM) of LMH001 and its breakdown products, compounds **13** and **15**, were diluted 10-fold in PBS and transferred to a 384-well plate (5 µL per well). The assay was performed in a volume of 50 µL per well in transparent non-binding surface 384-well plates (Corning #3640). Differentiated PLB-985 cells (10^6^ cells/mL) were resuspended in HBSS buffer (GIBCO #14025050) supplemented with 1.6 mM WST1 (1 mM final, Dojindo) and dispensed into the 384-well plate containing serial dilutions of compounds for 10 min. NOX2 was activated by addition of phorbol-12-myristate-13-acetate PMA (10 nM final). Absorbance (OD440) was measured with a Spectramax Paradigm reader (Molecular Devices) for 3 h. To graph the data, the rate of superoxide was calculated for the first hour. The flavoprotein inhibitor diphenyleneiodonium (DPI) and the NOX2 small molecule inhibitor GSK2795039 (Hirano et al., 2015) were used as controls.

## Results

### Synthesis of LMH001

**FIGURE 2:**
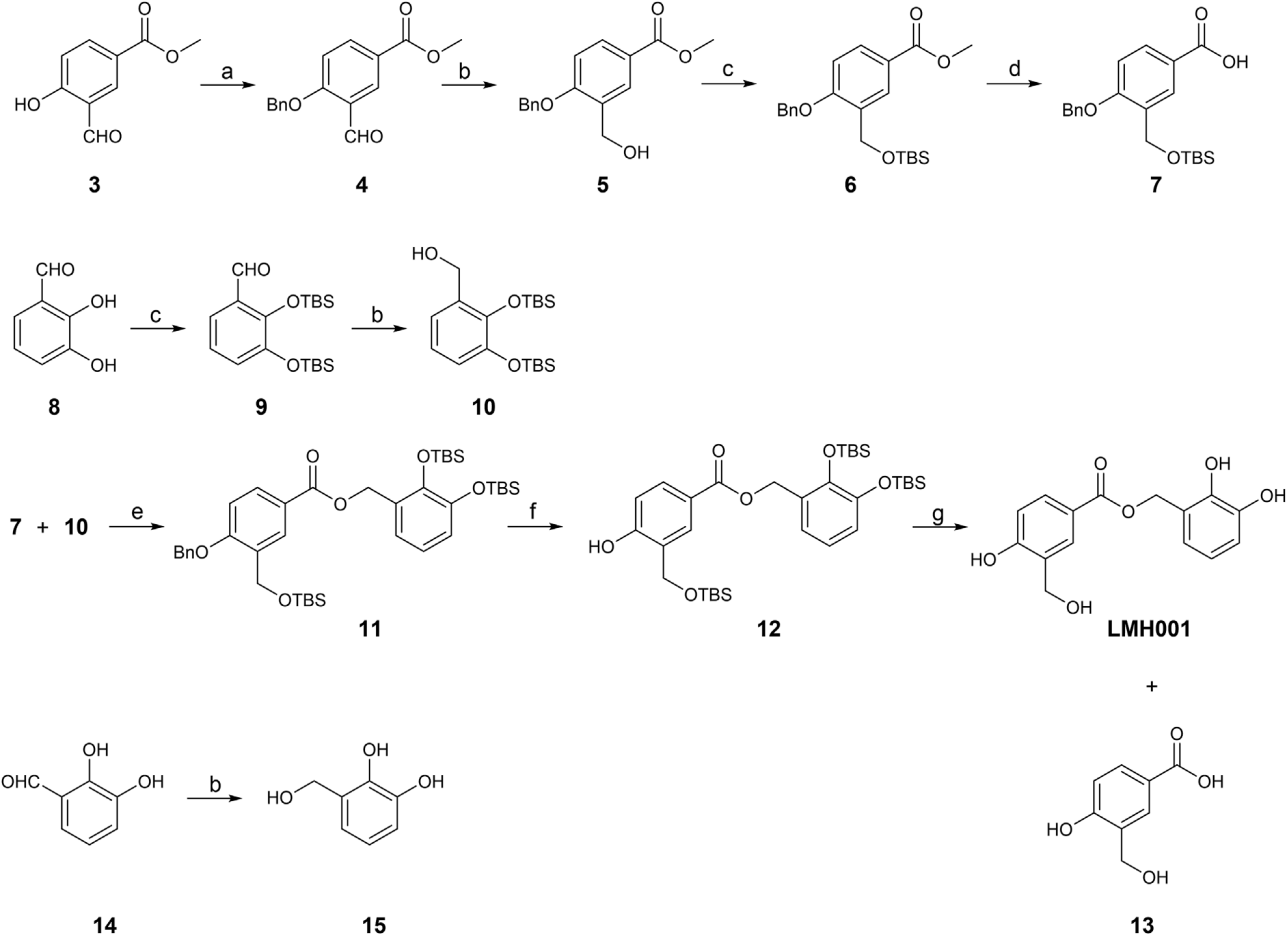
Synthetic scheme of LMH001 and its two breakdown products (**13** and **15**). Reagents and conditions: (a) benzyl bromide, Cs_2_CO_3_, anhydrous DMF, rt, 7.5 h, quantitative; (b) NaBH_4_, MeOH, 0°C or 0°C to rt, 12 h, 5% for **15** and quantitative for **5** and **10** (c) TBSCl, imidazole, DCM, rt, 12 h, 64%–73% (for **6**, **9**); (d) DCM, 3 M NaOH in MeOH, rt, 18 h, 66%; (e) DCC, DMAP, DCM, rt, 24 h, 60%; (f) H_2_ (1 atm), 10% Pd/C, MeOH, rt, 1 h, 40%; (g) 1 M TBAF in THF, THF, 0°C to rt, 1 h, 6%.

In Fan et al., LMH001 was obtained by custom synthesis from Tocris Biosciences (Bristol, United Kingdom) with a reference to the authors’ patent application describing LMH001 and other bi- and tri-aromatic compounds as NOX2 inhibitors (Li et al., 2013). The patent application contains a synthetic scheme for LMH001 and a brief description of the synthesis route, but no detailed procedures or characterization data. A protected acid intermediate and alcohol intermediate were prepared separately before an esterification followed by removal of the protection groups provided LMH001 in 10 steps. However, the *tert*-butyldimethylsilyl (TBS) protection group of the aromatic alcohol of the acid intermediate was cleaved off during the saponification and a re-protection step had to be introduced (Li et al., 2013). Instead, we developed a new 9-step synthetic route for LMH001, based on a more robust and orthogonal protection group strategy (Figure 2). The synthesis of key acid intermediate **7** began from **3**, which was subjected to benzyl group protection, aldehyde reduction, TBS protection, and saponification to afford **7** in good yield. The key alcohol intermediate **10** was produced by TBS protection of **8**, followed by aldehyde reduction of **9**, as described (Li et al., 2013). LMH001 was obtained after coupling reaction between **7** and **10**, followed by deprotection of the benzyl and TBS groups. During the purification of LMH001 by normal-phase chromatography, one of the LMH001 breakdown products, the acidic compound **13**, was obtained as a byproduct. The corresponding alcohol breakdown product, compound **15**, was synthesized by reduction of 2,3-dihydroxybenzaldehyde **14** with sodium borohydride (Figure 2).

### Binding activity and stability of LMH001

**FIGURE 3:**
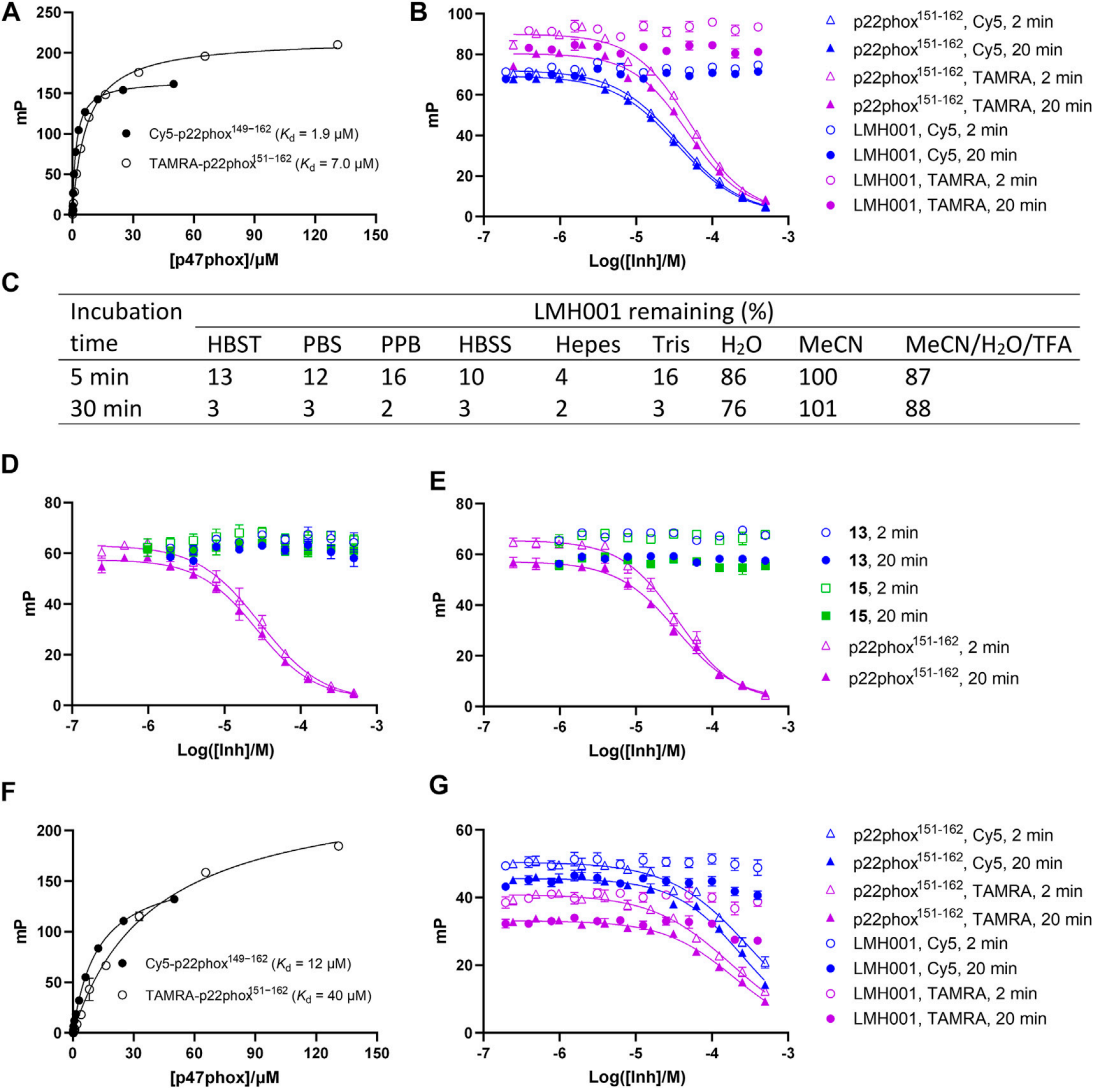
**(A–B and D–G)** Interaction of LMH001 and its breakdown products (**13**, **15**) with p47phox^SH3A–B^ measured by FP. The binding constant (*K*_d_) between p47phox^SH3A–B^ and Cy5-p22phox^149–162^ or TAMRA-p22phox^151–162^ in 1×HBST **(A)** or water **(F)**. Inhibition of the p47phox^SH3A–B^/Cy5-p22phox^149–162^ or p47phox^SH3A–B^/TAMRA-p22phox^151–162^ interaction with LMH001 in 1×HBST **(B)** or water **(G)** over 2 min or 20 min incubation. The unlabeled p22phox^151–162^ peptide was used as positive control. In 1×HBST, p22phox^151–162^ showed a *K*_i_ value of 14–22 µM when using Cy5-p22phox^149–162^ and a *K*_i_ value of 17–31 µM when using TAMRA-p22phox^151–162^ over 2–20 min. In water, p22phox^151–162^ showed a *K*_i_ value of 170–220 µM when using Cy5-p22phox^149–162^ and a *K*_i_ value of 140–155 µM when using TAMRA-p22phox^151–162^ over 2–20 min **(C)** LC–MS stability studies of LMH001 over time (5, 30 min) in different buffers. The percent remaining is measured by comparing AUC (UV250) of the LMH001 peaks in buffers at 5 or 30 min with AUC (UV250) of LMH001 in MeCN at 5 min. Inhibition of the p47phox^SH3A–B^/Cy5-p22phox^149–162^ **(D)** or p47phox^SH3A–B^/TAMRA-p22phox^151–162^ **(E)** interaction by **13** or **15** in the FP assay using 1×HBST and 2 or 20 min incubation.

The FP assay is a common method for studying PPI inhibitors (Milroy et al., 2014; Tran et al., 2019). By adding increasing concentrations of test compound to a fixed amount of a small fluorescent probe and the protein of interest, the dissociation constant of the interaction between the inhibitor and protein is determined as a *K*_i_ value. We here tested LMH001 against peptide probes (Cy5-p22phox^149−162^ and TAMRA-p22phox^151−162^) based on the PRR of p22phox and their interaction with the tandem SH3 domain of p47phox (p47phox^SH3A−B^), as done previously (Solbak et al., 2020). LMH001 did not show any activity in the FP assay up to 400 µM (Figures 3A, B). This was independent on the probe (Cy5 or TAMRA-based) or incubation time (2 or 20 min), and in contrast to the non-labelled p22phox^151−162^ control peptide that showed *K*_i_ values similar to those reported previously (Solbak et al., 2020).

This result was surprising, as LMH001 was shown to clearly inhibit the interaction between GST-p47phox^SH3A−B^ and a FITC-labelled p22phox^151-162^ peptide similar to our probes in the original paper and with high affinity (IC_50_ = 0.149 µM, *K*_i_ = 0.054 µM) (Fan et al., 2022). To investigate this discrepancy, we conducted several experiments. First, LMH001 is an ester (Figure 1B), which is generally susceptible to chemical hydrolysis in water and buffers or sensitive to enzymatic degradation in biological fluids (de Souza et al., 2022). We therefore tested the stability of LMH001 in the FP assay buffer—a standard Hepes based buffer with sodium chloride and tween 20 at physiological pH (HBST). LMH001 degraded very fast with only 13% and 3% left after 5 and 30 min, respectively (Figure 3C and Supplementary Figure S1). The same result was obtained using other standard and physiological relevant assay buffers, such as PBS, PPB, HBSS, Hepes, or Tris (Figure 3C and Supplementary Figure S1). Interestingly, LMH001 was more stable in pure water, with 86% and 76% left after 5 and 30 min, respectively, and even more stable in pure MeCN and an acidic mix of water/MeCN with 0.1% TFA (Figure 3C). Finally, it was observed that degradation in the HBST buffer increased with pH going from 6 to 8 (Supplementary Figure S2).

The expected degradation process of an ester in aqueous buffers is hydrolysis, which would lead to the carboxylic acid and alcohol containing breakdown products - corresponding to compound **13** and **15** for LMH001 (Figure 2). Accordingly, compounds with masses similar to **13** and **15** were clearly detected in the LC−MS chromatograms during the stability tests. We also synthesized **13** and **15** enabling us to confirm by LC−MS that indeed compound **13** and **15** are the breakdown products of LMH001 (Supplementary Figure S1).

We next tested if the breakdown products of LMH001 were active in the FP assay when tested as isolated compounds. However, this was not the case (Figures 3D, E). Also, performing the FP assay in pure water, in which LMH001 is reasonably stable, did not result in any measurable activity of LMH001 (Figures 3F, G). We then considered if assay artifacts such as fluorescence inner-filter effects could explain the apparent activity of LMH001 in the FP assay reported by Fan et al. The probes we use in FP are based on the red-shifted fluorophores Cy5 and TAMRA. These fluorophores are generally more advantageous for avoiding false-positive signals caused by fluorescence interferences compared to fluorophores emitting in the green area around 520 nM, such as fluorescein (Turek-Etienne et al., 2004; Simeonov et al., 2008) or FITC as used in the FP probe by Fan et al. However, LMH001 did not affect the fluorescence intensity of the red-shifted probes used in FP nor of a short FITC-labelled peptide (Supplementary Figure S3). Based on this, there are no indications of LMH001 giving rise to fluorescent artifacts that can be interpreted as activity.

Structurally, the catechol moiety of LMH001 is a cause of concern as such groups can oxidize to protein-reactive quinones and produce radicals through redox cycling; hence, compounds containing the catechol substructure are flagged as so-called pan-assay interference compounds (PAINS) (Baell and Holloway, 2010). Also, LMH001 shares structural similarity to phenolic Mannich bases, which can form protein-reactive quinone methides (McLean et al., 2009; Baell and Holloway, 2010). We therefore investigated by LC−MS if LMH001 covalently reacts with p47phox^SH3A−B^, as this could perhaps explain some of the activities reported by Fan et al. Interestingly, incubating LMH001 in a 17-fold molar excess relative to p47phox^SH3A−B^ led to a protein mass increase of 122 Da corresponding to a reaction involving a quinone methide intermediate (Supplementary Figure S4). However, in contrast to ebselen, which fully converted p47phox^SH3A−B^ to its molecular adduct, only about 20%–30% of p47phox^SH3A−B^ was modified by LMH001 in HBST and negligible conversion was observed in water (Supplementay Figure S4).

### Cellular activity of LMH001

**FIGURE 4:**
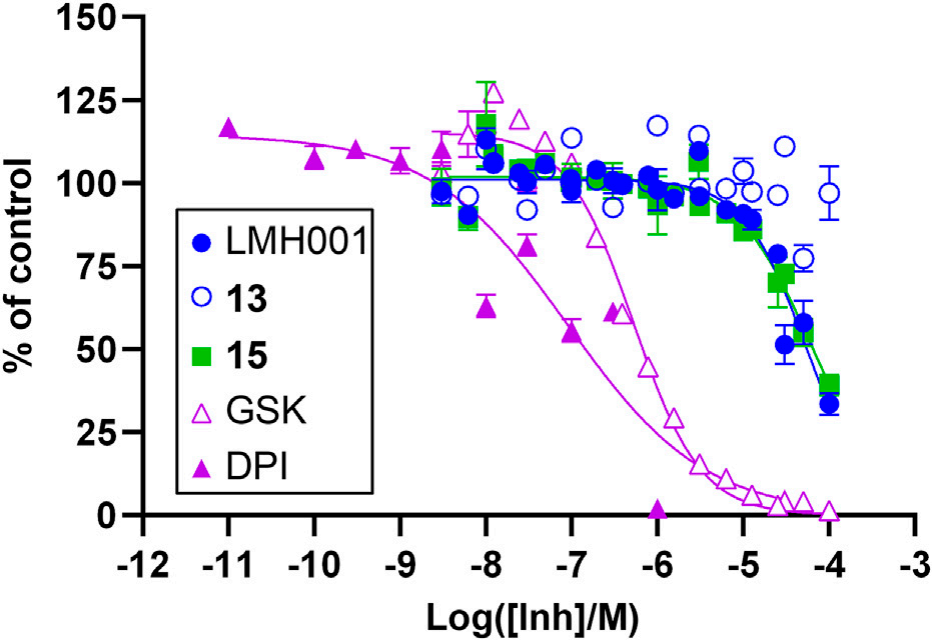
Inhibitory effects of LMH001 (IC_50_ = 54 μM), **13**, **15** (IC_50_ = 63 μM) and the control compounds GSK2795039 (GSK; IC_50_ = 0.53 μM) and DPI (IC_50_ = 0.090 μM) in the NOX2 cell-based assay. Data for two independent experiments each performed in triplicates are shown as mean ± SEM.

In order to evaluate a potential inhibitory effect of LMH001 and the two breakdown products **13** and **15**, we performed concentration-dependent measurements of the impact of the compounds on NOX2-dependent superoxide generation by PLB-985 cells stimulated with the phorbol ester PMA (Supplementary Figure S5 and Figure 4). LMH001 showed weak NOX2 inhibitory activity (IC_50_ = 54 µM), approximately 200 times less potent than the activity observed by Fan et al. in COS-phox cells (0.24 µM). Interestingly, the breakdown product **15** showed an inhibition pattern (IC_50_ = 63 µM) globally overlapping with LMH001, while the other breakdown product **13** displayed no activity. Overall, this suggests that the weak measured NOX2 inhibition of LMH001 is due to the breakdown product **15**.

## Discussion

Chemical compounds that activate or inhibit the activity of disease-relevant proteins are valuable tools for target validation during the drug discovery process and can be used as lead compounds. However, the value as a probe for studying biological events depends, among other things, on the reliability of the data and specificity of the compound (Arrowsmith et al., 2015; Blagg and Workman, 2017). Many NOX2 inhibitors appeared promising at first glance, but later proved to be unspecific or false positives (Wind et al., 2010; Zielonka et al., 2014; Augsburger et al., 2019; Reis et al., 2020), as also seen in other fields (Tran et al., 2019).

LMH001 was designed by molecular docking to bind in the pocket formed by the SH3A−B domain of p47phox that interacts with the PRR of p22phox. Accordingly, LMH001 was shown to be able to inhibit the p47phox^SH3A−B^/p22phox PPI in FP (Fan et al., 2022). The effect of LMH001 in decreasing endothelial NOX2 activity and angiotensin II-induced vascular oxidative stress in mice (but not p47phox knockout mice) was attributed to this mechanism (Fan et al., 2022). These results were very encouraging and considering the lack of reliable and potent chemical probes for studying p47phox/p22phox inhibition, LMH001 could provide significant progress to the field of NOX2 drug discovery and pharmacology.

Unfortunately, our FP results clearly showed that LMH001 was unable to inhibit the p47phox^SH3A−B^/p22phox PPI in FP, in contrast to the previously published data (Fan et al., 2022). Furthermore, because the compound was degraded within minutes in standard assay buffers, the FP assay had to be performed in pure water in order to test the affinity of LMH001 itself. This degradation could be caused by hydrolysis of the ester bond in LMH001 resulting in breakdown products **13** and **15**. These were also inactive in the FP assay. In an attempt to explain the discrepancy between our FP data and those of Fan et al., we investigated if LMH001 could cause fluorescence inner-filter effects to the probes (based on Cy5, TAMRA, and FITC), but this was not the case. Instead, considering that LMH001 contains substructures shared by PAINS, such as a catechols and phenolic Mannich bases, we tested if LMH001 covalently reacted with p47phox^SH3A−B^. Indeed, LMH001 led to partial conversion into a modified protein with an increased molecular weight (MW) corresponding to a Mannich base-like reaction *via* a quinone methide intermediate of LMH001. Although, this covalent reaction apparently did not functionally affect the interaction between p47phox^SH3A−B^ and the p22phox peptide probe in our FP assay, it may induce effects in assays using other p47phox protein constructs or in cells.

There could also be other explanations for the disagreement between our FP data and those of Fan et al. Perhaps LMH001 was not made in pure form in the original study and an impurity was responsible for the apparent activity. Of notice, chemical characterization data for compound identity and purity are lacking in the paper by Fan et al. Alternatively, a very specific buffer might be needed for obtaining chemical stability of LMH001 and activity in the FP assay. Fan et al. did not specify the assay buffer. Notably, molecular docking used by Fan et al. to design LMH001 cannot count as evidence for binding (Gimeno et al., 2019), and the potential interaction between LMH001 and p47phox has not been characterized by biophysical methods–such as SPR, isothermal titration calorimetry (ITC), or NMR–which would otherwise be able to demonstrate direct ligand-protein binding (Renaud et al., 2016).

Despite the lack of evidence for LMH001 being able to bind p47phox, its instability in buffers, and the ambiguous FP data, LMH001 might still have genuine pharmacological effects. Alternative mechanisms could be antioxidant properties, inhibition of NOX2 by other ways than binding into the p47phox^SH3A−B^ pocket, or activity *via* other proteins than p47phox or even NOX2. However, since LMH001 is a chemically unstable molecule, any pharmacological effect it may have is likely due to degradation products or metabolites. In Fan et al., a very short half-life of 0.042 h (2.5 min) was reported after iv injection in mice, supporting this notion. Fan et al. observed that LMH001 was >200-fold more potent in inhibiting superoxide produced by NOX2 in cells activated by PMA (IC_50_ = 0.24 µM), compared to antioxidant quenching of superoxide produced in a cell-free xanthine oxidase assay. In our cell assay, however, LMH001 only demonstrated weak NOX2 inhibitory activity (IC_50_ = 54 µM), which is in the potency-range of the antioxidant effects reported by Fan et al. Interestingly, the catechol breakdown product **15** showed similar inhibitory activity as LMH001. This indicates that the weak NOX2 inhibition of LMH001 was in fact due to the compound degrading to **15** causing antioxidant activity.

Another interesting aspect of LMH001 and its breakdown products **13** and **15** is the structural similarity to apocynin (Figure 1B; Figure 2). Apocynin is a methoxy-substituted catechol originally shown to reduce superoxide production by activated neutrophils *via* inhibiting the translocation of cytoplasmic NOX2 subunits. This effect depends on the cellular presence of myeloperoxidases, which converts apocynin to its dimeric form (diapocynin) or even its trimer (Simons et al., 1990; Stolk et al., 1994; Ximenes et al., 2007). Apocynin has been frequently used in the literature as a NOX2 inhibitor, but several studies have demonstrated reproducibility issues and lack of selectivity to NOX isoforms due to direct antioxidant and off-target effects (Wind et al., 2010; Cifuentes-Pagano et al., 2014; Altenhofer et al., 2015; Hirano et al., 2015; Augsburger et al., 2019). Numerous off-target effects of apocynin have been reported. For example, apocynin inhibits Rho kinase activity, which contributed to vasodilation *in vitro* (Schluter et al., 2008). Similarly, apocynin, vanillin (an apocynin analogue), and their dimeric forms inhibit focal adhesion kinase (FAK) and Akt kinase activity (Jantaree et al., 2017). NOX2 activity is reduced by inhibition of kinases such as Akt (Chen et al., 2003; Hoyal et al., 2003). Vanillic acid also has a broad range of pharmacological effects, including inhibition of c-jun n-terminal kinases (JNK) (Ullah et al., 2020). An alternative on-target mechanism suggested for apocynin (Ximenes et al., 2007; Mora-Pale et al., 2012; Jiang et al., 2013), is that peroxidase metabolism creates reactive quinones that react with cysteine residues in p47phox leading to inhibition of NOX2 complex assembling. Interestingly, the trimeric quinone form of apocynin reacts with Cys196 of p47phox, the same cysteine that ebselen reacts with (Solbak et al., 2020), and thereby disrupt the p47phox/p22phox interaction (Mora-Pale et al., 2012). Overall, perhaps LMH001 under certain conditions works as a prodrug that after degradation, metabolism, or dimerization/trimerization indirectly inhibits NOX2 *via* some of the mechanisms reported for apocynin and its analogues.

In conclusion, we were not able to reproduce the FP data reported by Fan et al. Our data suggest that LMH001 does not bind p47phox^SH3A−B^ or inhibits the p47phox^SH3A−B^/p22phox PPI. Furthermore, LMH001 is highly unstable in standard buffers and is thereby in practice an untestable compound. The main degradation products **13** and **15** were also inactive in FP. Consequently, the reported pharmacological activity of LMH001 (Fan et al., 2022) cannot be ascribed to binding into the p47phox^SH3A−B^ pocket by either LMH001 or its breakdown products. Weak NOX2 inhibition was observed for LMH001 in cells, but this was likely caused by degradation into **15** and resulting antioxidant activity. We also noticed the structural similarity of LMH001 and the degradation products **13** and **15** with apocynin–a well-known but pharmacologically very complicated and unspecific molecule. Based on this, we hypothesize that LMH001 might work under some biological conditions as a prodrug for apocynin-like compounds. Overall, LMH001 is in our view not a useful chemical probe for investigating NOX2’s role in biology until its mechanism of action is elucidated in details.

## Data Availability

The original contributions presented in the study are included in the article/Supplementary Material, further inquiries can be directed to the corresponding author.
