# Supplementary material for: Chemical synthesis of a reported p47phox/p22phox inhibitor and characterization of its instability and irreproducible activity
Source: Front Pharmacol. 2023 Jan 5;13:1075328. doi: 10.3389/fphar.2022.1075328 (PMC9850094; doi:10.3389/fphar.2022.1075328)
Supplement: Supplementary file 1 [file DataSheet1.pdf]

frontiers

Supplementary Material

Chemical Synthesis of a Reported p47phox/p22phox Inhibitor and Characterization of its Instability and Irreproducible Activity

Jie Zang[1], Yves Cambet[2], Vincent Jaquet[2,3], Anders Bach[1*]

[1]Department of Drug Design and Pharmacology, Faculty of Health and Medical Sciences, University of Copenhagen, Copenhagen, Denmark

[2]READS unit**,** Centre Médical Universitaire, University of Geneva, Geneva, Switzerland

[3]Department of Pathology and Immunology, Centre Médical Universitaire, University of Geneva, Geneva, Switzerland

*E-mail: anders.bach@sund.ku.dk

Synthesis of LMH001

General Procedures. All chemicals used for synthesis were obtained from commercial suppliers and used without prior purification. ^{1}H NMR, ^{13}C NMR, and 2D NMR (^{1}H−^{1}H COSY, ^{1}H−^{13}C HSQC, ^{1}H−^{13}C HMBC) spectra were recorded using either a 600 MHz Bruker Avance III HD instrument equipped with a cryogenically cooled 5 mm dual probe or a 400 MHz Bruker Avance III instrument equipped with a 5 mm broad band probe. Samples were dissolved in either DMSO-*d*$_6$ (VWR Chemicals, 99.80% D) or $CDCl_3$ (Cambridge Isotope Laboratories, Inc., 99.8% D) and analyzed at 300 K. Thin layer chromatography (TLC) analyses were performed using TLC silica gel 60 F_{254} aluminum plates (Merck). Liquid chromatography−mass spectrometry (LC–MS) spectra were obtained using the same system as described for the stability studies. Flash column chromatography was carried out using either prepacked RediSep Rf silica flash cartridges or a RediSep Rf reversed-phase C18 cartridge on a CombiFlash® Rf+ apparatus. Buffer A (milliQ H_2O:MeCN:TFA 95:5:0.1 v/v%) and buffer B (milliQ H_2O:MeCN:TFA 5:95:0.1 v/v%) were used for reverse-phase chromatography. All final and tested compounds showed ≥ 95% purity according to NMR and LC−MS.

*Methyl 4-(benzyloxy)-3-formylbenzoate (**4**).* The compound was synthesized according to a previously described procedure (Leung and Chow, 2017) with minor deviations. Benzyl bromide (2.00 g, 1.39 mL, 11.1 mmol) was added dropwise to a mixture of methyl 3-formyl-4-hydroxybenzoate (**3**, 1.00 g, 5.55 mmol) and Cs_2CO_3 (1.15 g, 8.33 mmol) in dry DMF (8.3 mL) at room temperature. The mixture was stirred until LC–MS showed full conversion of **3** (7.5 h). The reaction mixture was diluted with water and ethyl acetate (EtOAc). The aqueous phase was extracted three times with EtOAc. The combined organic layers were washed with water, brine, dried over Na_2SO_4, filtered and concentrated under reduced pressure. The residue was purified by normal-phase flash chromatography eluting with EtOAc in heptane (0–100%) to afford the desired product as a white solid (1.93 g, 7.14 mmol, quantitative yield). ^{1}H NMR (400 MHz, DMSO-*d*$_6$) δ 10.40 (s, 1H), 8.27 (d, *J* = 2.3 Hz, 1H), 8.20 (dd, *J* = 8.8, 2.4 Hz, 1H), 7.56 – 7.52 (m, 2H), 7.47 (d, *J* = 8.9 Hz, 1H), 7.45 – 7.40 (m, 2H), 7.39 – 7.34 (m, 1H), 5.39 (s, 2H), 3.85 (s, 3H). The ^{1}H NMR data matches the reported ppm values.

*Methyl 4-(benzyloxy)-3-(hydroxymethyl)benzoate (**5**)*. The compound was synthesized according to a previously described procedure (Luu and Li, 2022) with minor deviations. Compound **4** (1.93 g, 7.14 mmol) was dissolved in methanol (MeOH, 23.8 mL) and cooled to 0 °C. $NaBH_4$ (0.41 g, 10.71 mmol) was added in portions over 5 min. The mixture was stirred at 0 °C until LC–MS showed full conversion of **4** (12 h). The reaction was quenched by adding water while cooling on ice bath until no bubbles were formed. MeOH was removed under reduced pressure. The remaining aqueous phase was extracted with diethyl ether three times. The organic layers were combined, dried over Na_2SO_4, filtered and concentrated under reduced pressure. The desired product was obtained after concentration as a colorless oil (2.07 g, 7.60 mmol, quantitative yield) and used for the next step without further purification. ^{1}H NMR (400 MHz, DMSO-*d*$_6$) δ 8.05 (dt, *J* = 2.2, 1.0 Hz, 1H), 7.84 (dd, *J* = 8.6, 2.3 Hz, 1H), 7.48 – 7.44 (m, 2H), 7.40 (ddd, *J* = 7.6, 6.7, 1.4 Hz, 2H), 7.36 – 7.30 (m, 1H), 7.15 (d, *J* = 8.6 Hz, 1H), 5.23 – 5.19 (m, 3H), 4.59 – 4.54 (m, 2H), 3.82 (s, 3H). The ^{1}H NMR data matches with the reported value.

*Methyl 4-(benzyloxy)-3-(((tert-butyldimethylsilyl)oxy)methyl)benzoate (**6**)*. To a solution of **5** (2.07 g, 7.60 mmol) in dichloromethane (DCM, 7.6 mL) was added imidazole (1.04 g, 15.20 mmol) and *tert*-butyldimethylchlorosilane (1.43 g, 9.50 mmol). The resulting mixture was stirred at room temperature until LC–MS showed full conversion of **5** (12 h). The reaction mixture was filtered (suction) and the filter cake was rinsed with DCM. The filtrate was washed with water, dried over Na_2SO_4, filtered and concentrated under reduced

pressure. The crude was purified by normal-phase flash chromatography eluting with EtOAc in heptane (0–100%) to afford the desired product as a colorless oil (2.14 g, 5.54 mmol, 73%). ^{1}H NMR (400 MHz, DMSO-d_6) δ 8.03 (dd, *J* = 2.2, 1.1 Hz, 1H), 7.86 (dd, *J* = 8.6, 2.3 Hz, 1H), 7.48 – 7.43 (m, 2H), 7.43 – 7.37 (m, 2H), 7.37 – 7.30 (m, 1H), 7.17 (d, *J* = 8.6 Hz, 1H), 5.24 (s, 2H), 4.75 (s, 2H), 3.81 (s, 3H), 0.91 (s, 9H), 0.07 (s, 6H).

*4-(Benzyloxy)-3-(((tert-butyldimethylsilyl)oxy)methyl)benzoic acid (**7**)*. To a solution of **6** (2 g, 5.17 mmol) in DCM (46.5 mL) was added a methanolic solution of 3 M NaOH (5.17 mL, 16.51 mmol). The reaction mixture was stirred for 18 h. The solvent was removed under reduced pressure. The residue was diluted with water and washed with EtOAc. The aqueous phase was cooled, acidified to pH ~2 with 1 M HCl and extracted with EtOAc three tims. The combined organic layers were dried over Na_2SO_4, filtered and concentrated under reduced pressure. The crude was purified by normal-phase flash chromatography eluting with EtOAc in heptane (0–100%) to afford the desired product as a white solid (1.27 g, 3.41 mmol, 66%). ^{1}H NMR (400 MHz, DMSO-d_6) δ 12.54 (s, 1H), 8.01 (dd, *J* = 2.2, 1.1 Hz, 1H), 7.83 (dd, *J* = 8.6, 2.3 Hz, 1H), 7.48 – 7.43 (m, 2H), 7.42 – 7.37 (m, 2H), 7.36 – 7.31 (m, 1H), 7.13 (d, *J* = 8.6 Hz, 1H), 5.23 (s, 2H), 4.75 (d, *J* = 0.9 Hz, 2H), 0.91 (s, 9H), 0.07 (s, 6H).

*2,3-Bis((tert-butyldimethylsilyl)oxy)benzaldehyde (**9**).* The compound was synthesized according to a previously described procedure (Stein et al., 2018) with minor deviations. To a solution of 2,3-dihydroxybenzaldehyde (**8**, 1.00 g, 7.24 mmol) and imidazole (1.97 g, 29.0 mmol) in DCM (7.2 mL) was added *tert*-butyldimethylsilyl chloride (2.73 g, 18.1 mmol). The resulting mixture was stirred at room temperature until LC–MS showed full conversion of **8** (12 h). The reaction mixture was filtered (suction) and the filter cake was rinsed with DCM. The filtrate was washed with water, dried over Na_2SO_4, filtered and concentrated under reduced pressure. The crude was purified by normal-phase flash chromatography eluting with EtOAc in heptane (0–100%) to afford the desired product as a pale yellow oil (1.71 g, 4.66 mmol, 64%). ^{1}H NMR (400 MHz, DMSO-d_6) δ 10.26 (d, *J* = 0.8 Hz, 1H), 7.30 (dd, *J* = 7.8, 1.7 Hz, 1H), 7.22 (dd, *J* = 8.0, 1.7 Hz, 1H), 7.03 (td, *J* = 7.8, 0.8 Hz, 1H), 1.00 (s, 9H), 0.95 (s, 9H), 0.25 (s, 6H), 0.13 (s, 6H). The ^{1}H NMR data matches with the reported value.

*(2,3-Bis((tert-butyldimethylsilyl)oxy)phenyl)methanol (**10**)*. The compound was synthesized according to a previously described procedure (Stein et al., 2018) with minor deviations. To a solution of **9** (2.65 g, 7.23 mmol) in MeOH (36.2 mL) was added sodium borohydride (0.51 g, 13.48 mmol) at 0 °C. Then, the mixture was stirred at room temperature until LC–MS showed full conversion of **9** (12 h). The reaction was quenched by adding water while cooling on ice bath until no bubbles were formed. MeOH was removed under reduced pressure. The remaining aqueous phase was extracted with diethyl ether three times. The organic layers were combined, dried over Na_2SO_4, filtered and concentrated under reduced pressure. The desired product was obtained after concentration as a colorless oil (2.6 g, 7.06 mmol, 98%) and used for next step without further purification. ^{1}H NMR (400 MHz, DMSO-d_6) δ 7.01 (ddt, *J* = 7.6, 1.7, 0.8 Hz, 1H), 6.84 (t, *J* = 7.8 Hz, 1H), 6.75 (dd, *J* = 8.0, 1.7 Hz, 1H), 5.00 (t, *J* = 5.6 Hz, 1H), 4.47 (d, *J* = 5.6 Hz, 2H), 0.98 (s, 9H), 0.92 (s, 9H), 0.20 (s, 6H), 0.11 (s, 6H). The ^{1}H NMR data matches with the reported value.

*2,3-Bis((tert-butyldimethylsilyl)oxy)benzyl 4-(benzyloxy)-3-(((tert-butyldimethylsilyl)oxy)methyl)benzoate (**11**).* Compound **7** (1.27 g, 3.41 mmol), DMAP (0.042 g, 0.34 mmol), and **10** (1.26 g, 3.41 mmol) were dissolved in DCM (17 mL) at room temperature for 15 min. Then *N*,*N*'-dicyclohexylcarbodiimide (DCC, 1.06 g, 5.11 mmol) was added in portions to the reaction mixture. The resulted reaction mixture was stirred for 24 h, and then diluted with DCM and filtered through celite. The filtrate was washed with water, brine, dried over Na_2SO_4, filtered, and concentrated under reduced pressure. The residue was purified by normal-phase flash chromatography eluting with EtOAc in heptane (0–100%) to afford the desired product as a colorless oil (1.49

g, 2.06 mmol, 60%). ^{1}H NMR (600 MHz, DMSO-d_6) δ 8.04 (dd, *J* = 2.3, 1.1 Hz, 1H), 7.87 (dd, *J* = 8.6, 2.3 Hz, 1H), 7.47 – 7.44 (m, 2H), 7.41 – 7.37 (m, 2H), 7.36 – 7.31 (m, 1H), 7.18 (d, *J* = 8.7 Hz, 1H), 7.00 (dd, *J* = 6.8, 2.5 Hz, 1H), 6.91 – 6.86 (m, 2H), 5.26 – 5.23 (m, 4H), 4.73 (s, 2H), 0.97 (s, 9H), 0.93 (s, 9H), 0.86 (s, 9H), 0.23 (s, 6H), 0.14 (s, 6H), 0.04 (s, 6H).

*2,3-Bis((tert-butyldimethylsilyl)oxy)benzyl 3-(((tert-butyldimethylsilyl)oxy)methyl)-4-hydroxybenzoate (**12**).* To a solution of **11** (0.1 g, 0.14 mmol) in MeOH (2.8 mL) was added 10% Pd/C (10 mg). The reaction flask was degassed three times with H_2, charged with a H_2 balloon and stirred at room temperature for 1 h. The catalyst was removed by filtration through a pad of celite and the filtrate was concentrated under reduced pressure. The reside was purified by normal-phase flash chromatography eluting with EtOAc in heptane (0–100%) to afford the desired product as a colorless oil (35 mg, 0.055 mmol, 40%). ^{1}H NMR (400 MHz, Chloroform-*d*) δ 8.66 (s, 1H), 7.92 (dd, *J* = 8.5, 2.2 Hz, 1H), 7.71 (d, *J* = 2.1 Hz, 1H), 6.99 (t, *J* = 4.7 Hz, 1H), 6.88 (d, *J* = 8.5 Hz, 1H), 6.85 – 6.83 (m, 2H), 5.34 (s, 2H), 4.94 (s, 2H), 1.02 (s, 9H), 0.97 (s, 9H), 0.93 (s, 9H), 0.24 (s, 6H), 0.18 (s, 6H), 0.15 (s, 6H).

2,3-Dihydroxybenzyl 4-hydroxy-3-(hydroxymethyl)benzoate (LMH001). To a solution of **12** (35 mg, 0.055 mmol) in 1.1 mL of THF under argon at 0 °C, was added tetrabutylammonium fluoride (TBAF, 166 µL, 0.166 mmol, 1 M in THF), and the solution was stirred for 1 h. The reaction mixture was concentrated under reduced pressure. The residue was purified by normal-phase flash chromatography eluting with MeOH in DCM (0–10%) and reverse-phase chromatography eluting with a gradient of Buffer B (0–100%) in Buffer A to afford the desired product as a white solid (1 mg, 0.0034 mmol, 6%). ^{1}H NMR (600 MHz, DMSO-d_6) δ 10.29 (s, 1H), 9.37 (s, 1H), 8.57 (s, 1H), 7.97 (dd, *J* = 2.3, 1.1 Hz, 1H), 7.71 (dd, *J* = 8.4, 2.3 Hz, 1H), 6.85 (d, *J* = 8.4 Hz, 1H), 6.78 – 6.75 (m, 2H), 6.63 (t, *J* = 7.8 Hz, 1H), 5.23 (s, 2H), 4.47 (s, 2H). ^{13}C NMR (151 MHz, DMSO) δ 165.83, 158.59, 145.10, 143.67, 129.40, 128.96, 128.66, 123.22, 120.23, 119.81, 118.77, 115.25, 114.38, 61.45, 57.68. LC–MS (ESI): m/z 289.0 $[M-1]^-$, t_R = 3.21 min.

*4-hydroxy-3-(hydroxymethyl)benzoic acid (**13**).* Compound **13** was isolated as a side product from the reaction making LMH001 from **12** during the normal-phase flash chromatography procedure and was further purified by reverse-phase chromatography (white solid, 6.1 mg). ^{1}H NMR (600 MHz, DMSO-d_6) δ 12.32 (s, 1H), 10.18 (s, 1H), 7.94 (s, 1H), 7.69 – 7.65 (m, 1H), 6.82 (d, *J* = 8.4 Hz, 1H), 5.07 (s, 1H), 4.48 (s, 2H). ^{13}C NMR (151 MHz, DMSO) δ 167.41, 158.27, 129.41, 128.97, 128.67, 121.08, 114.19, 57.78. LC–MS (ESI): m/z 167.1 $[M-1]^-$, t_R = 2.06 min.

*3-(hydroxymethyl)benzene-1,2-diol (**15**)*. The compound was synthesized according to a previously described procedure (Stein et al., 2018) with minor deviations. 2,3-Dihydroxybenzaldehyde (**14**, 0.20 g, 1.45 mmol) was dissolved in 1:1 MeOH/THF (4.8 mL) and cooled to 0 °C. $NaBH_4$ (0.082 g, 2.17 mmol) was added in portions over 5 min. The mixture was allowed to warm to room temperature and stirred for 4 h. The reaction was quenched by adding water while cooling on ice bath until no bubbles were formed. The mixture was concentrated under reduced pressure. The residue was purified by normal-phase flash chromatography eluting with MeOH in DCM (0–10%) and reverse-phase chromatography eluting with a gradient of Buffer B (0–100%) in Buffer A to afford the desired product as a white solid (9.5 mg, 0.068 mmol, 5%). ^{1}H NMR (600 MHz, DMSO-d_6) δ 9.07 (s, 1H), 8.18 (s, 1H), 6.74 (dd, *J* = 7.5, 1.7 Hz, 1H), 6.65 (dd, *J* = 7.9, 1.7 Hz, 1H), 6.59 (t, *J* = 7.7 Hz, 1H), 4.88 (t, *J* = 5.7 Hz, 1H), 4.47 (d, *J* = 5.5 Hz, 2H). ^{13}C NMR (151 MHz, DMSO-d_6) δ 144.54, 142.09, 129.28, 118.48, 117.86, 113.69, 58.43. LC–MS (ESI): m/z 139.0 $[M-1]^-$, t_R = 1.62 min.

NMR and LC–MS spectra of LMH001, 13, and 15

^{1}H NMR of LMH001

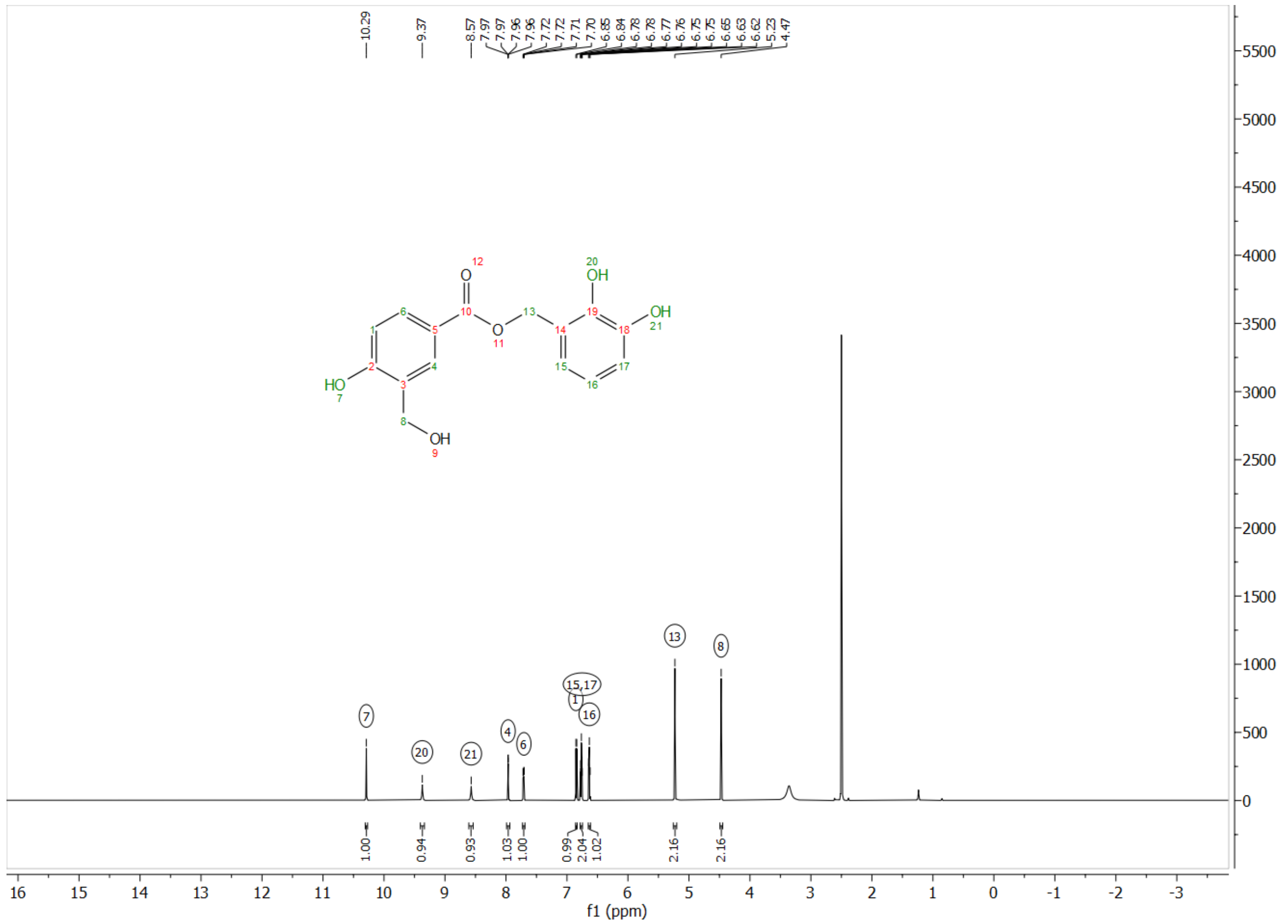

^{13}C NMR of LMH001

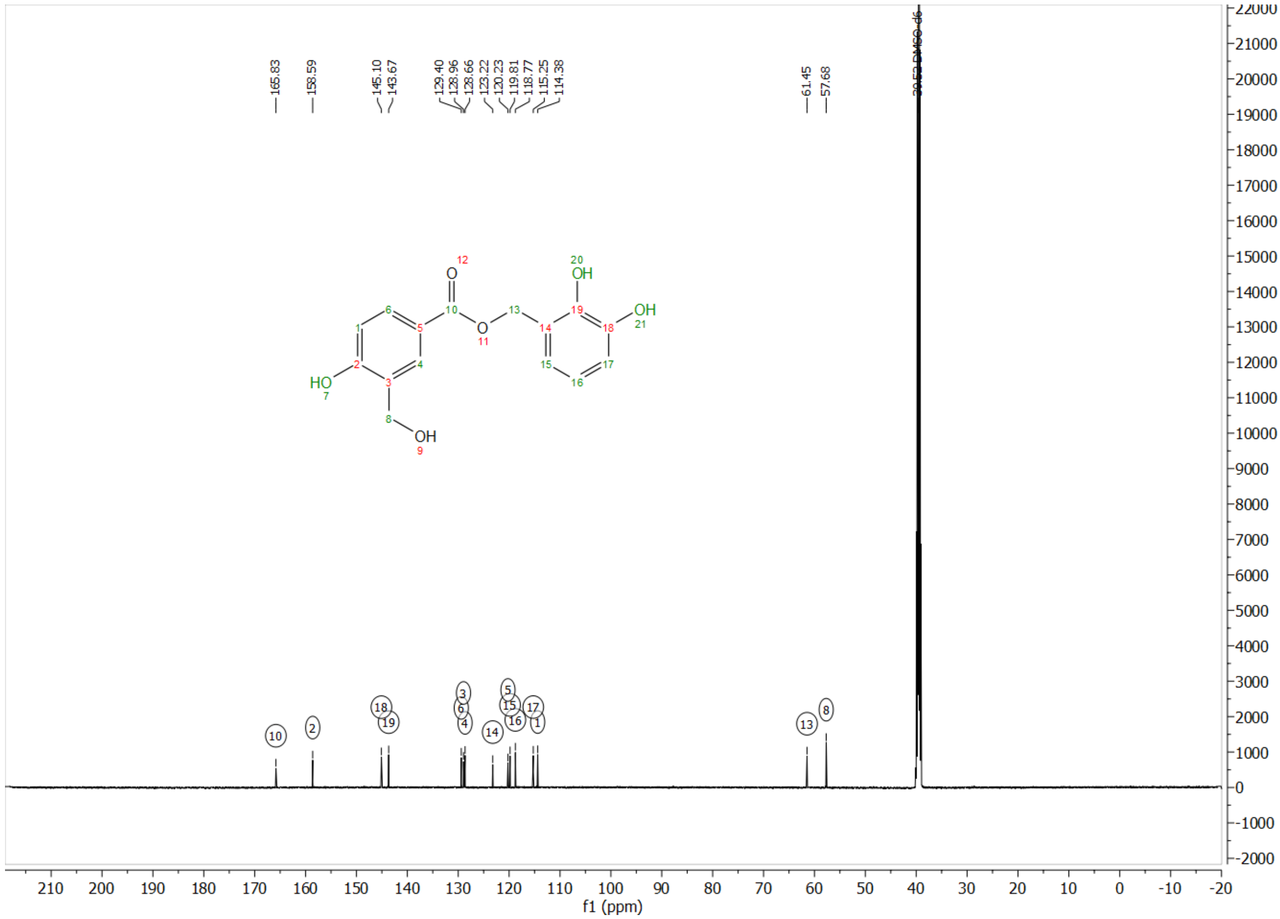

^{1}H-^{1}H COSY of LMH001

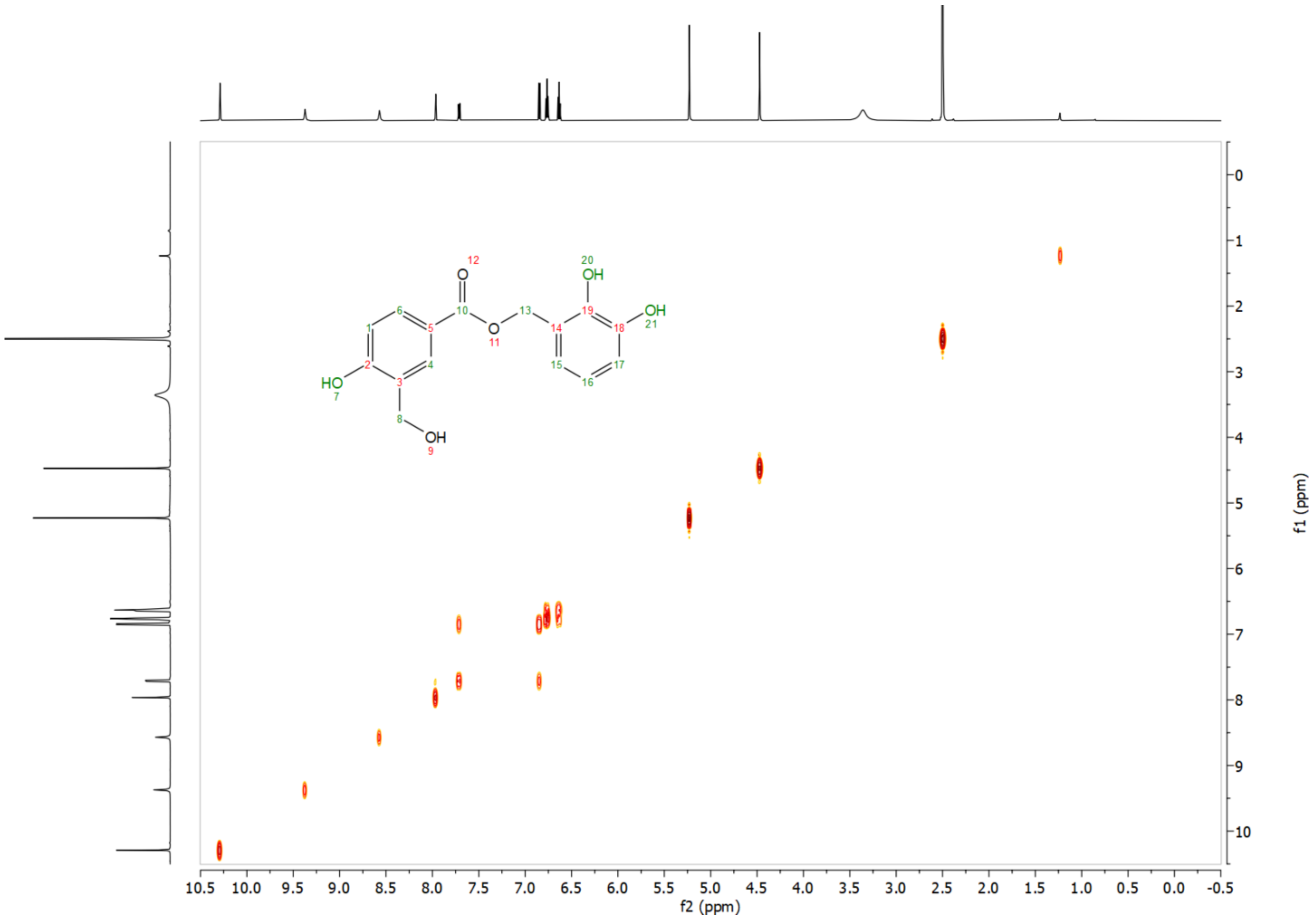

^{1}H-^{13}C HSQC of LMH001

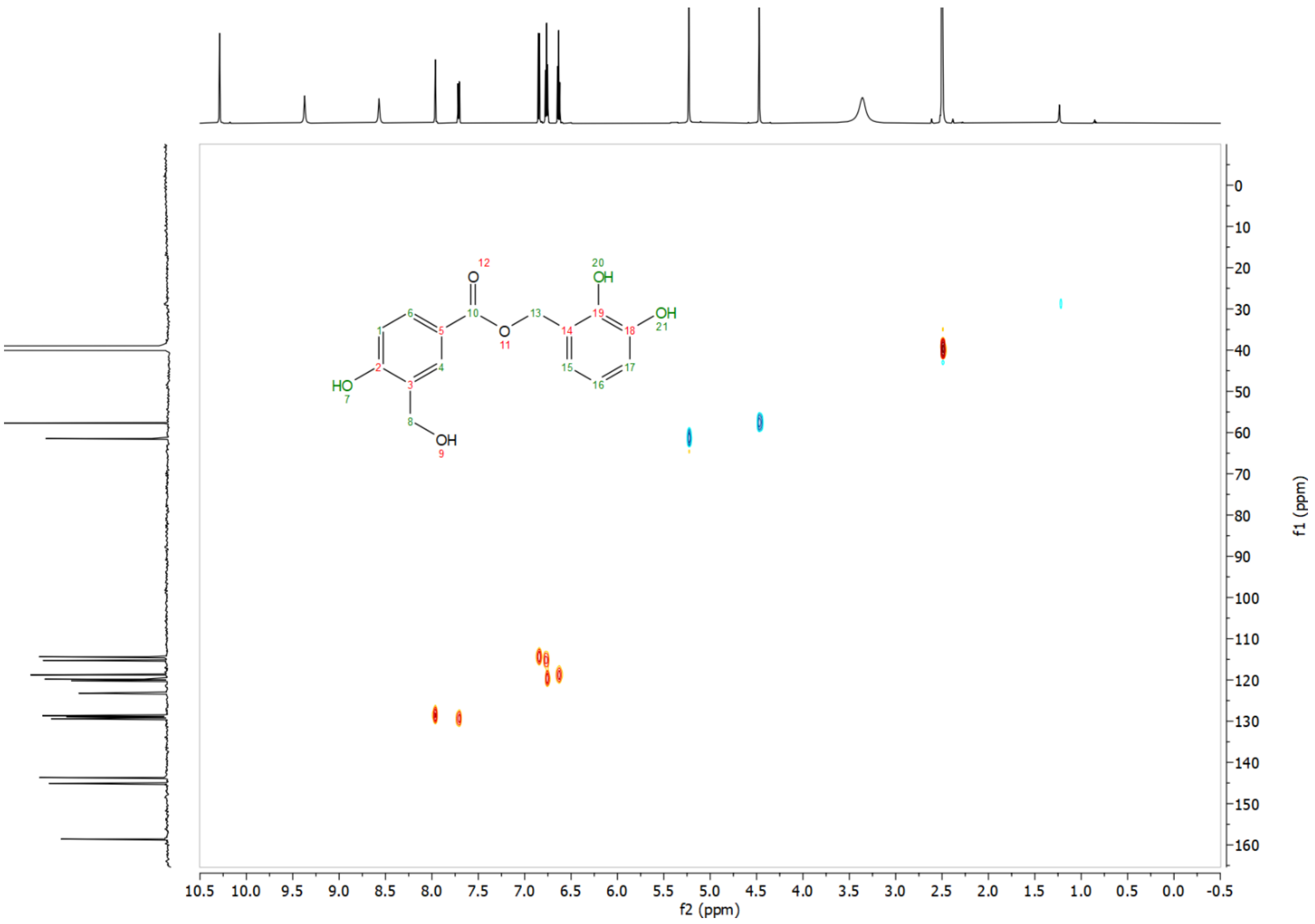

^{1}H-^{13}C HMBC of LMH001

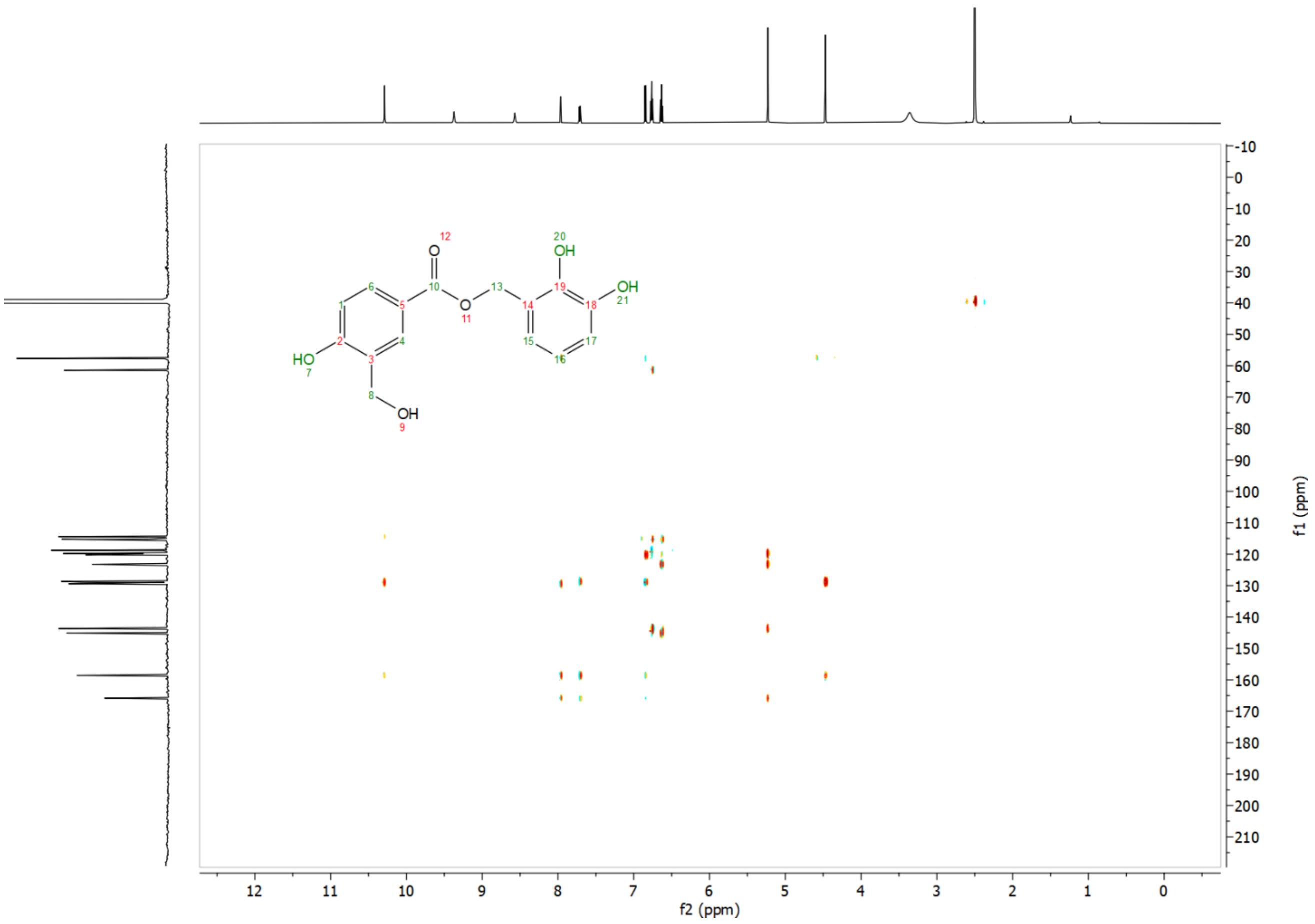

LC–MS spectra of LMH001

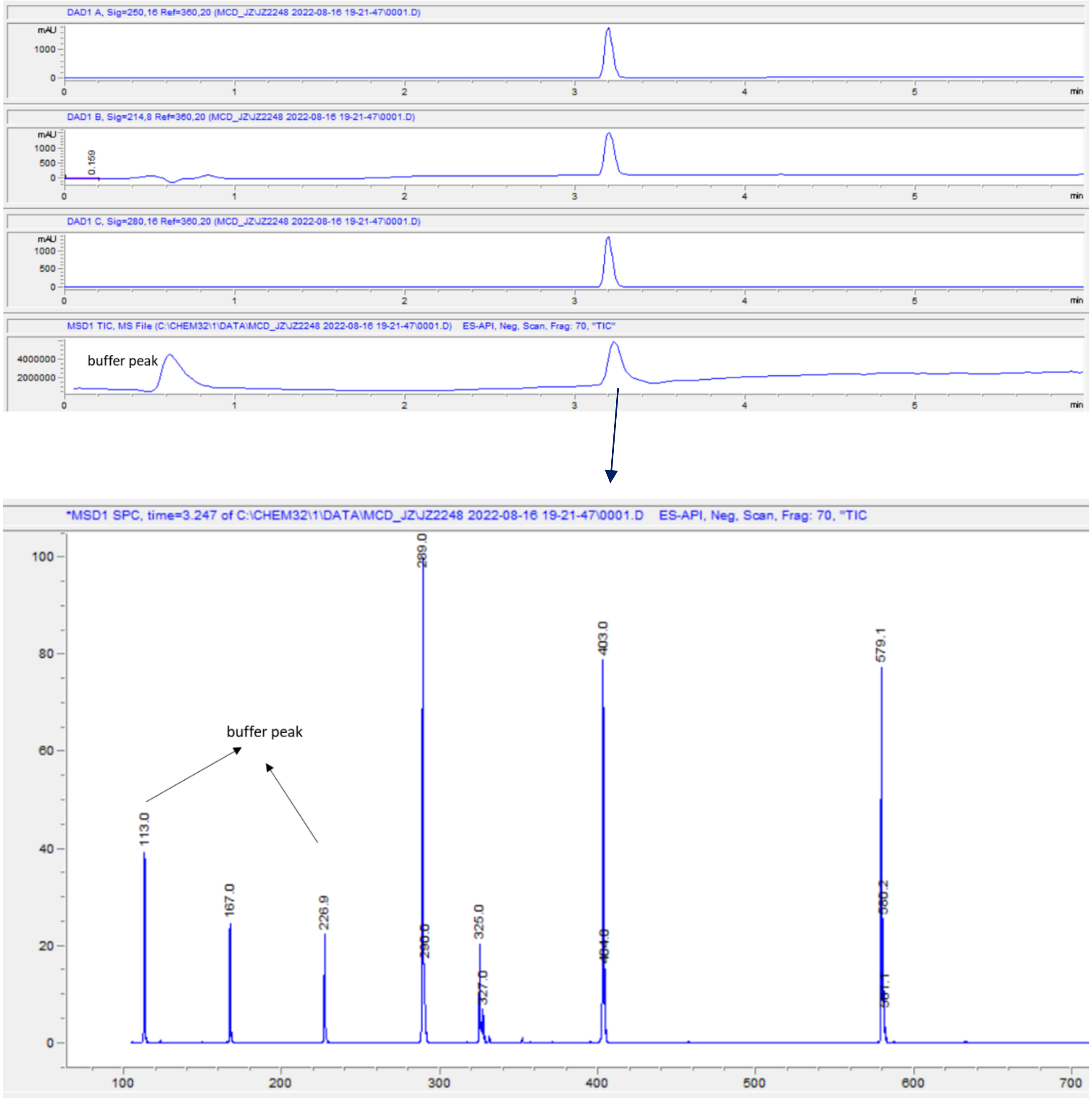

^{1}H NMR of compound **13**

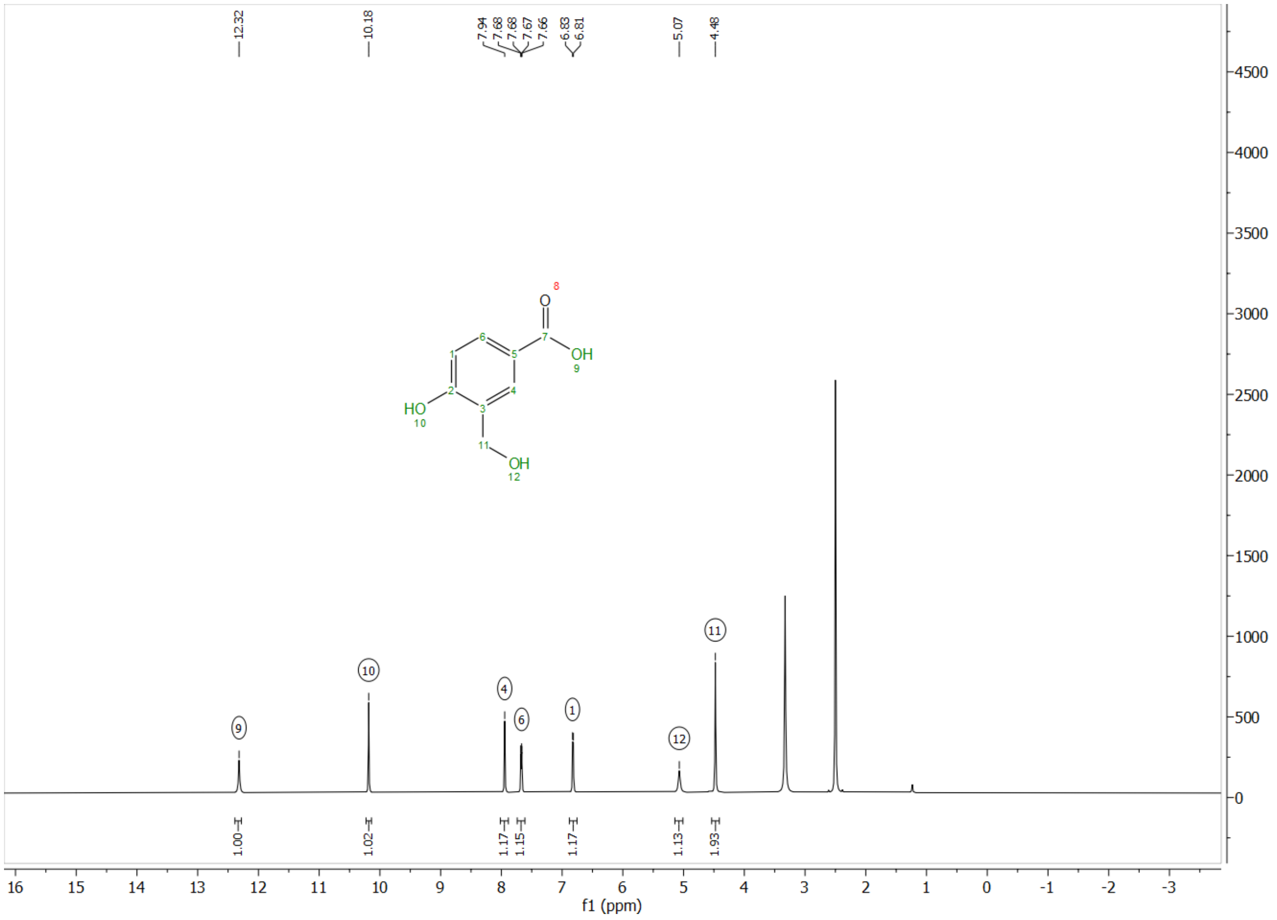

^{13}C NMR of compound **13**

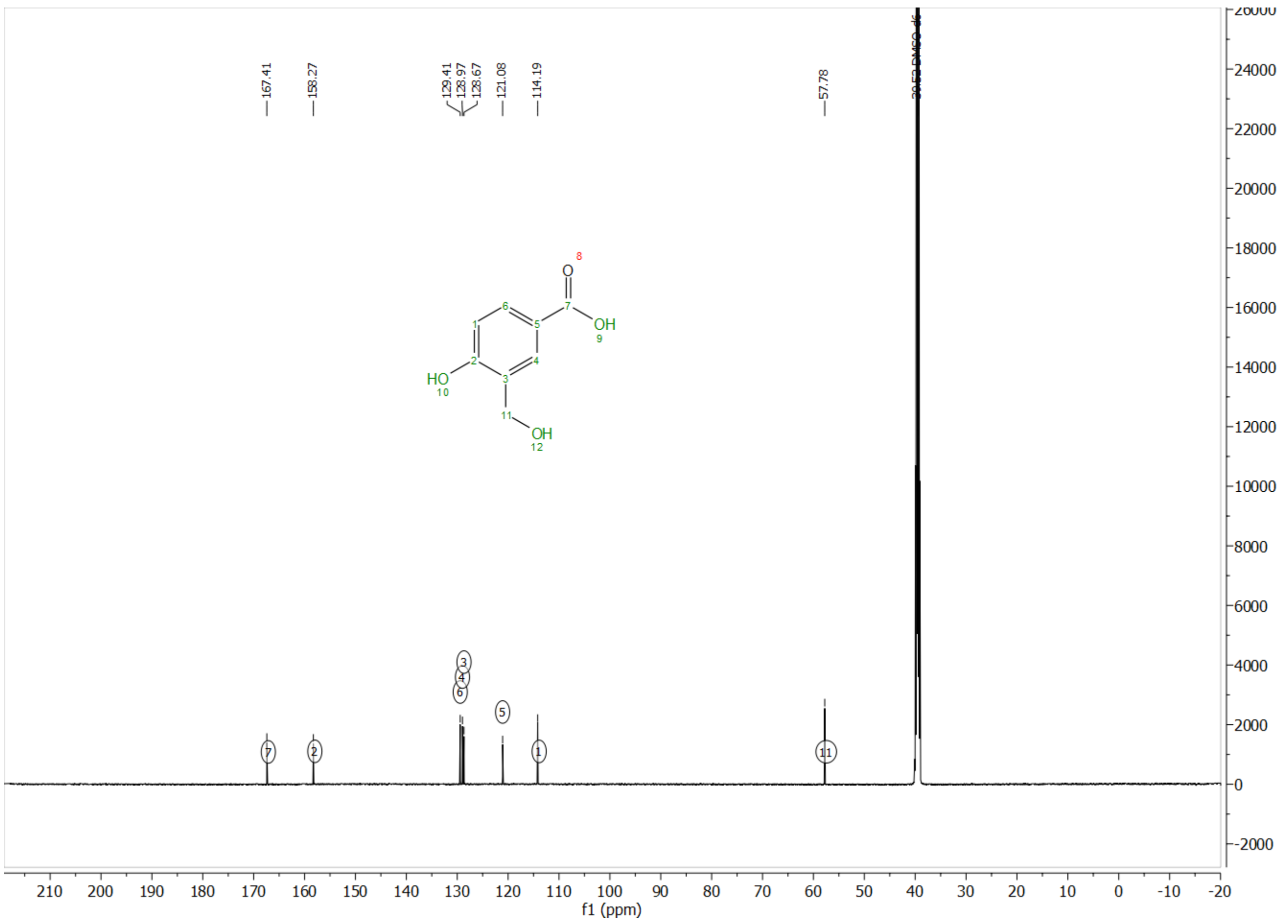

^{1}H-^{1}H COSY of **13**

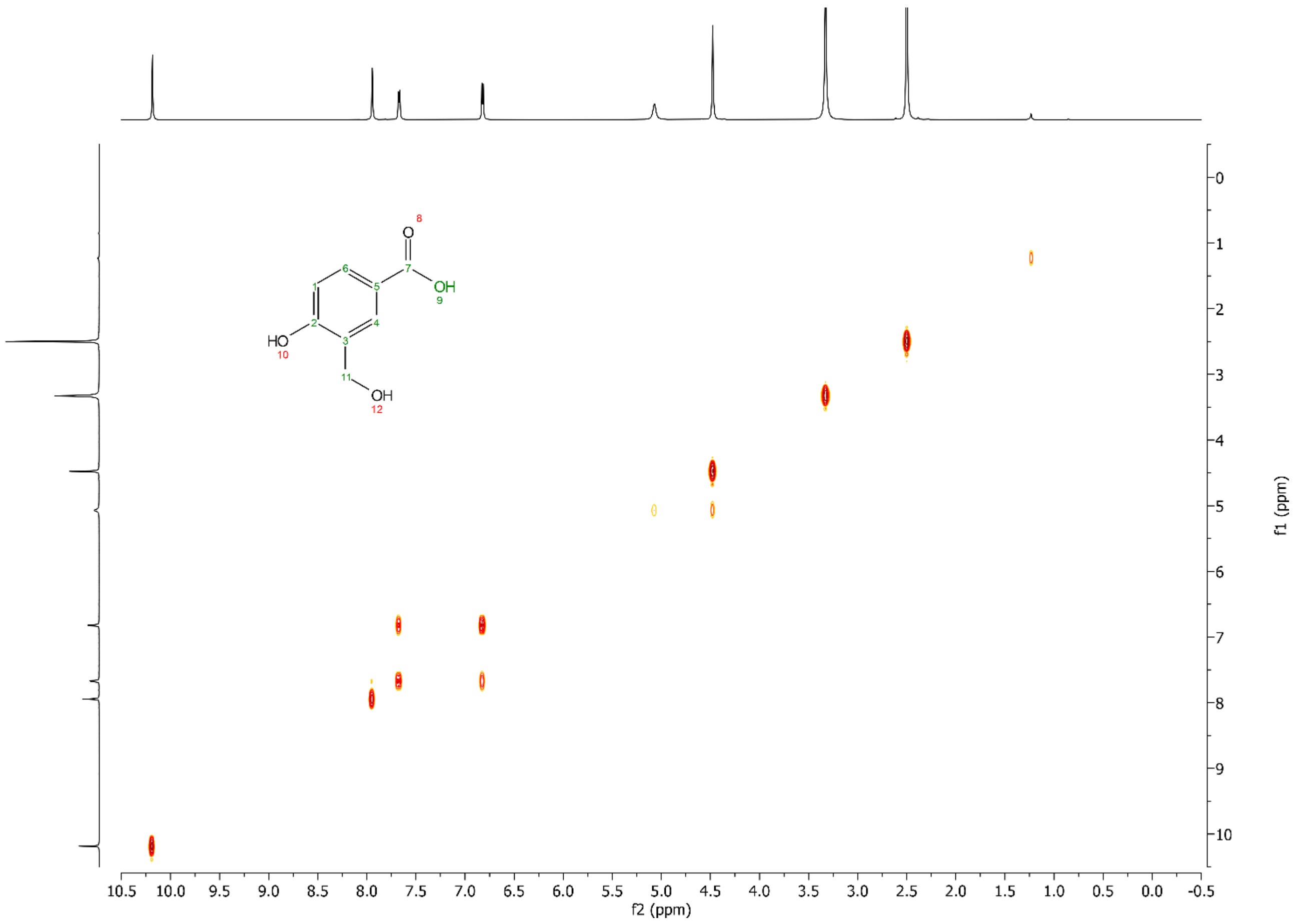

^{1}H-^{13}C HSQC of **13**

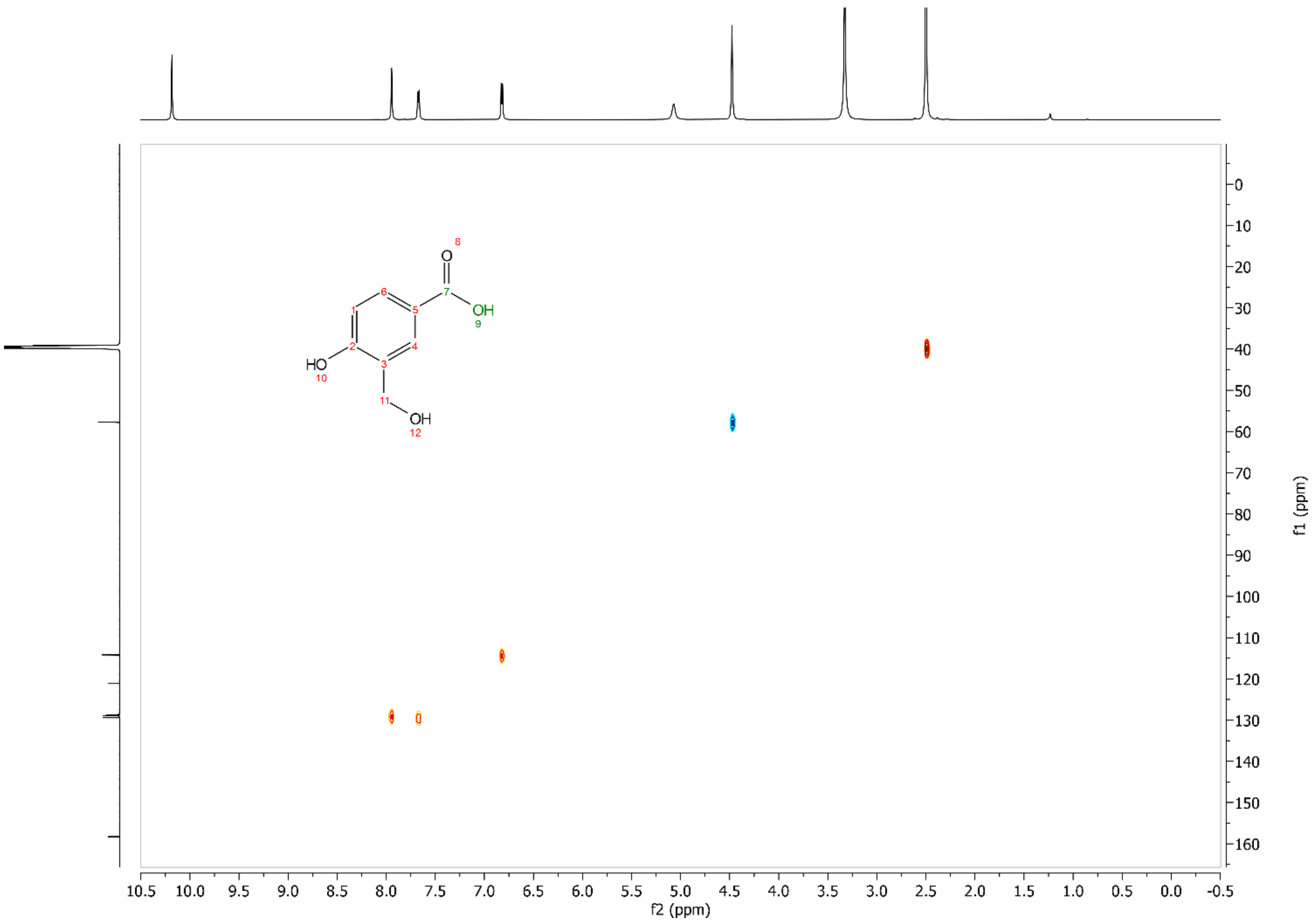

^{1}H-^{13}C HMBC of **13**

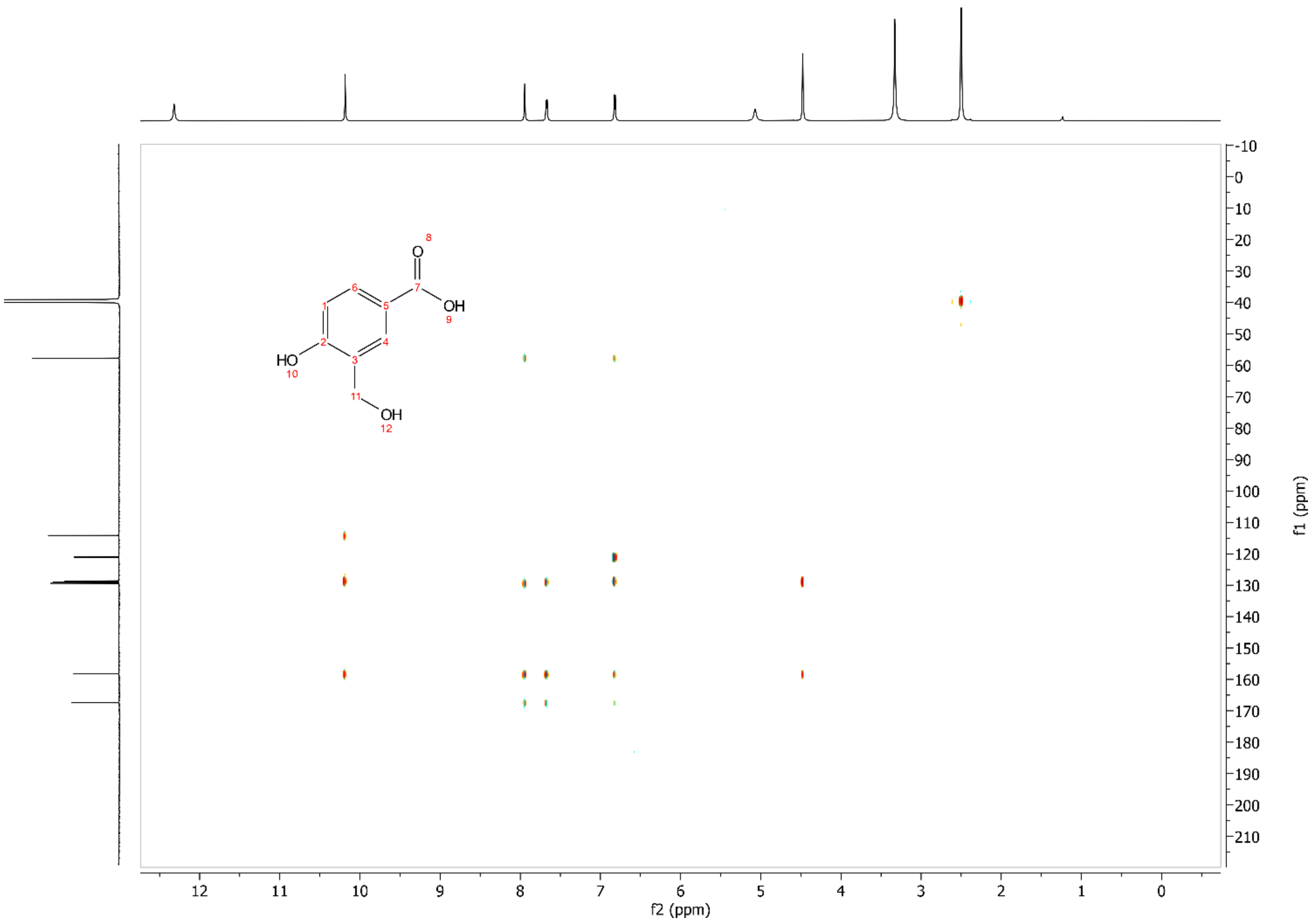

LC–MS spectra of **13**

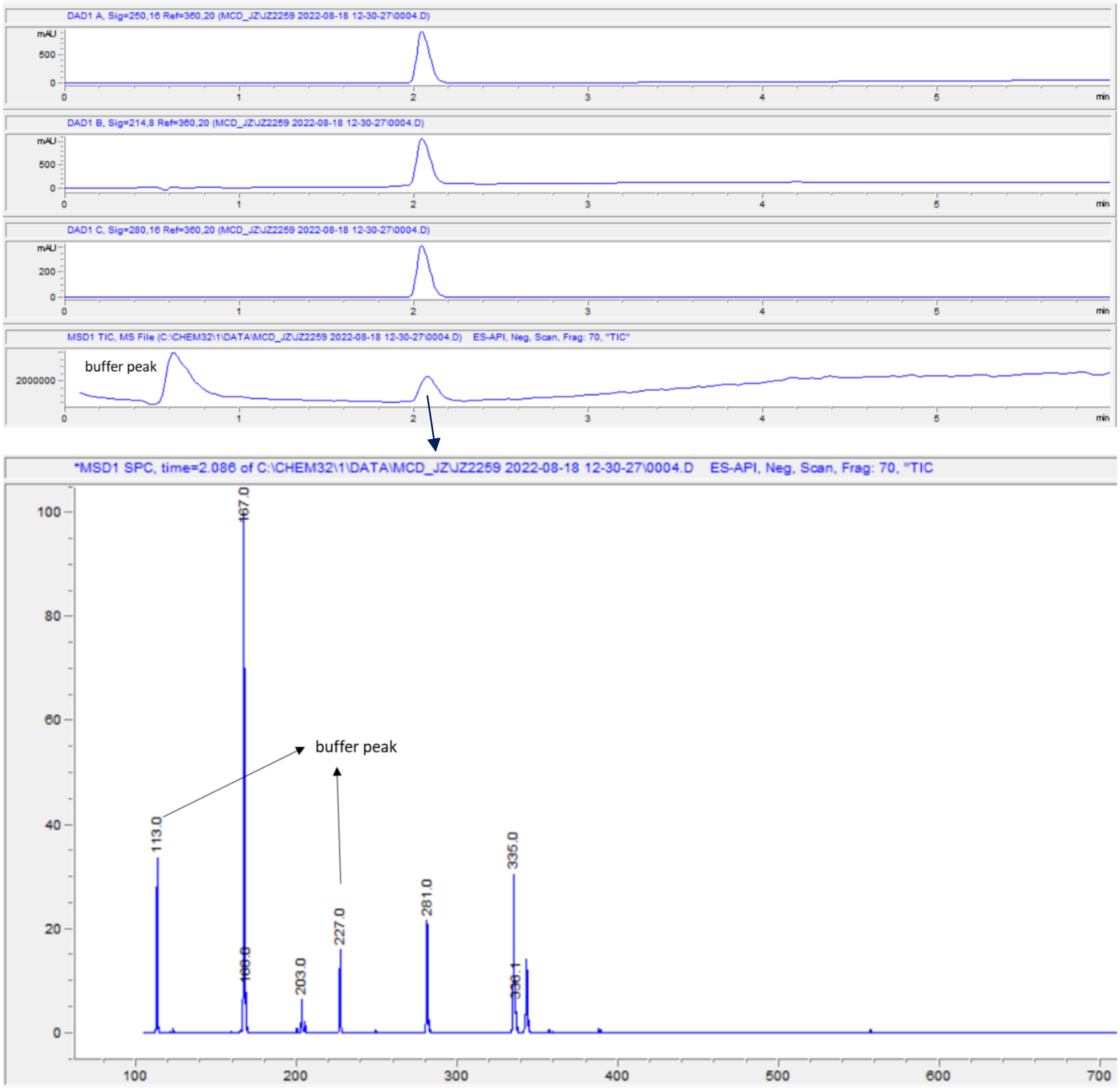

^{1}H NMR of compound **15**

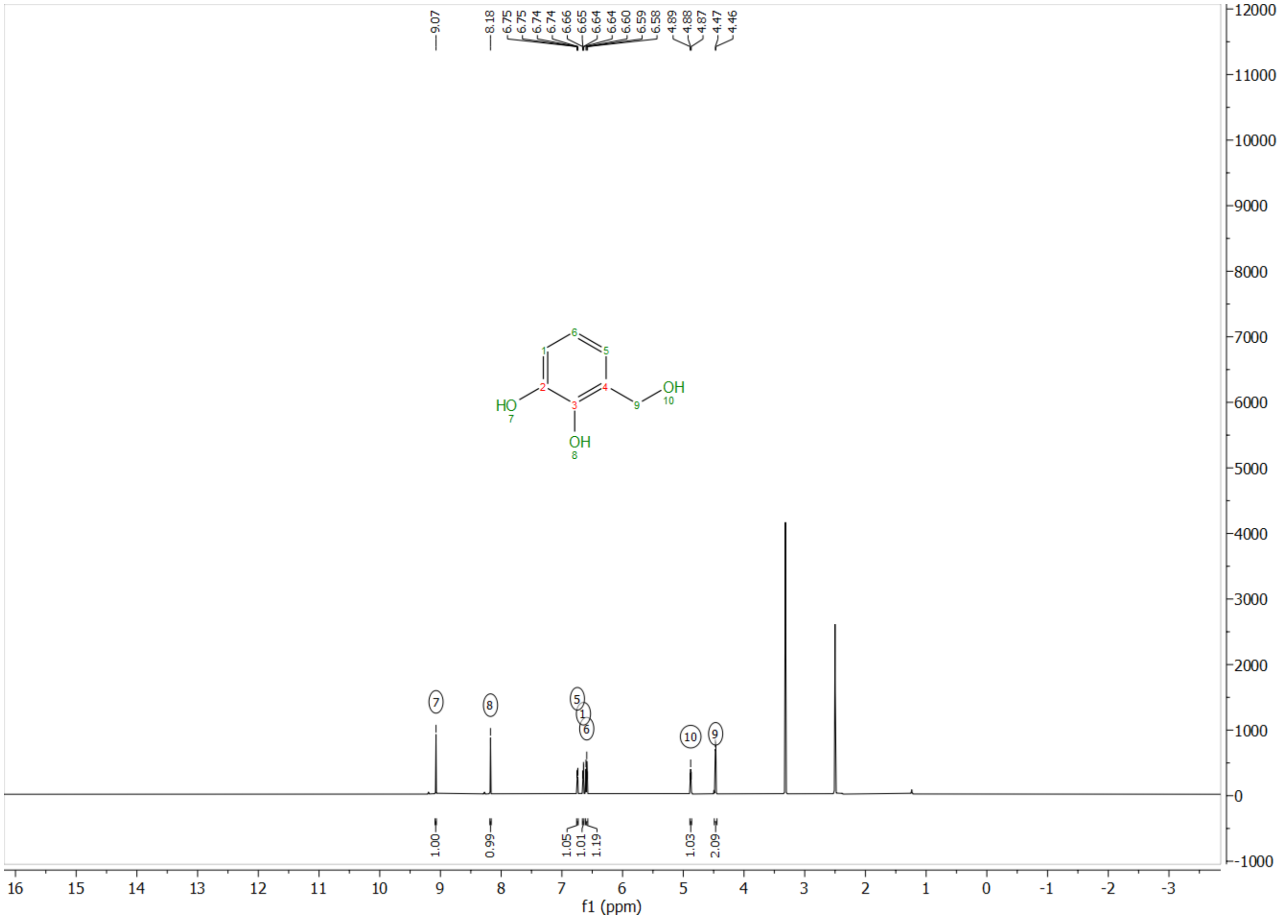

^{13}C NMR of compound **15**

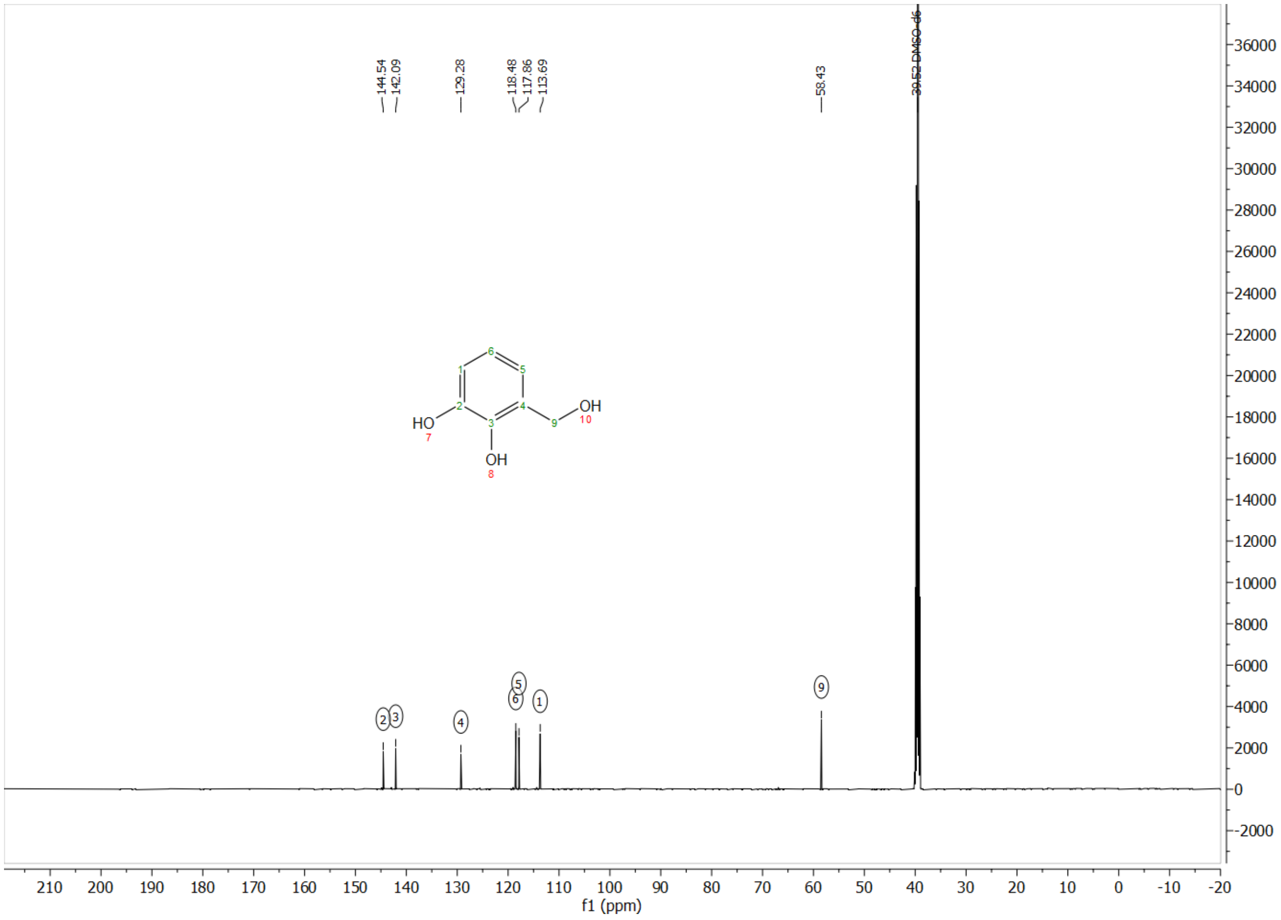

^1H-^1H COSY of **15**

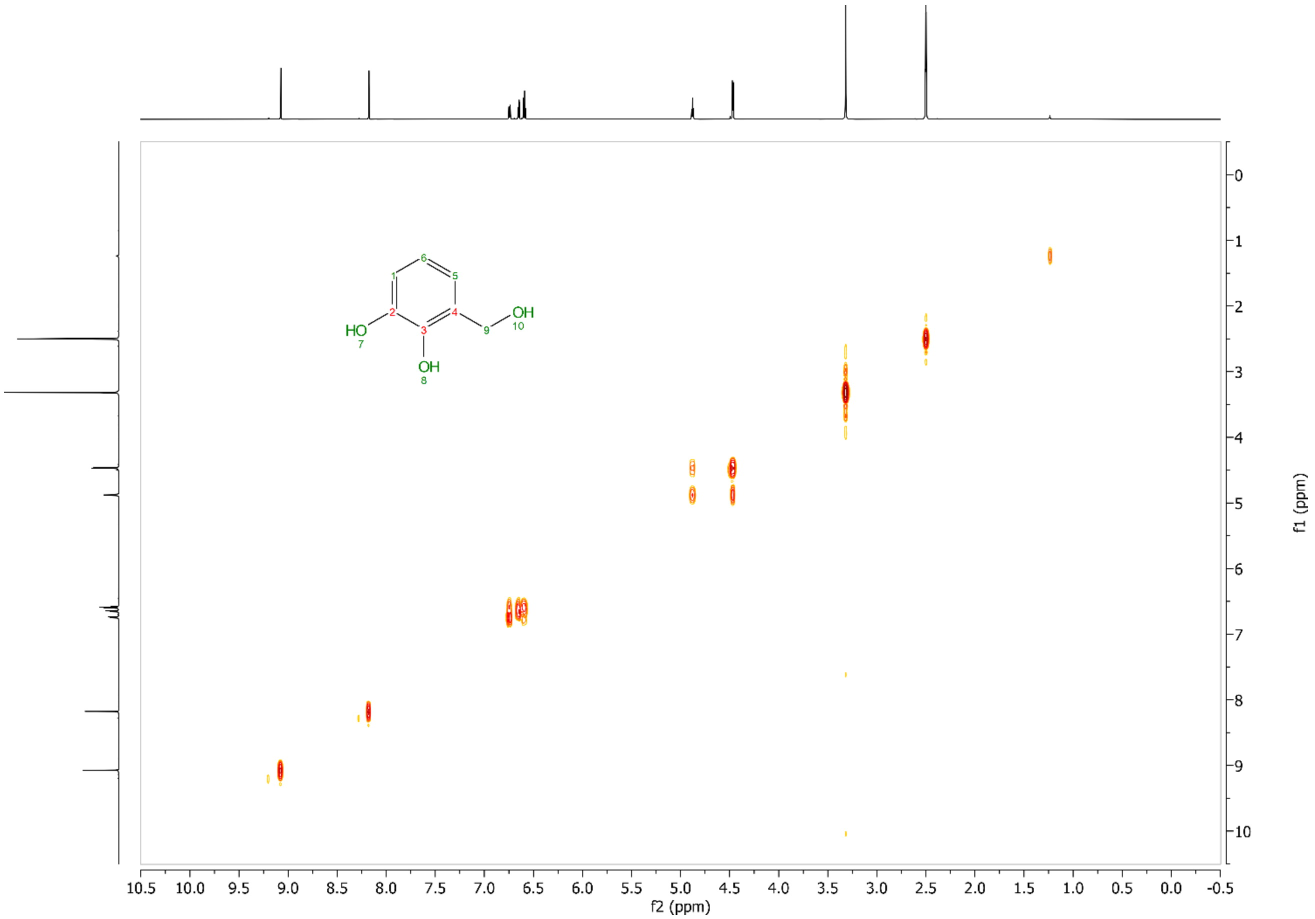

^{1}H-^{13}C HSQC of **15**

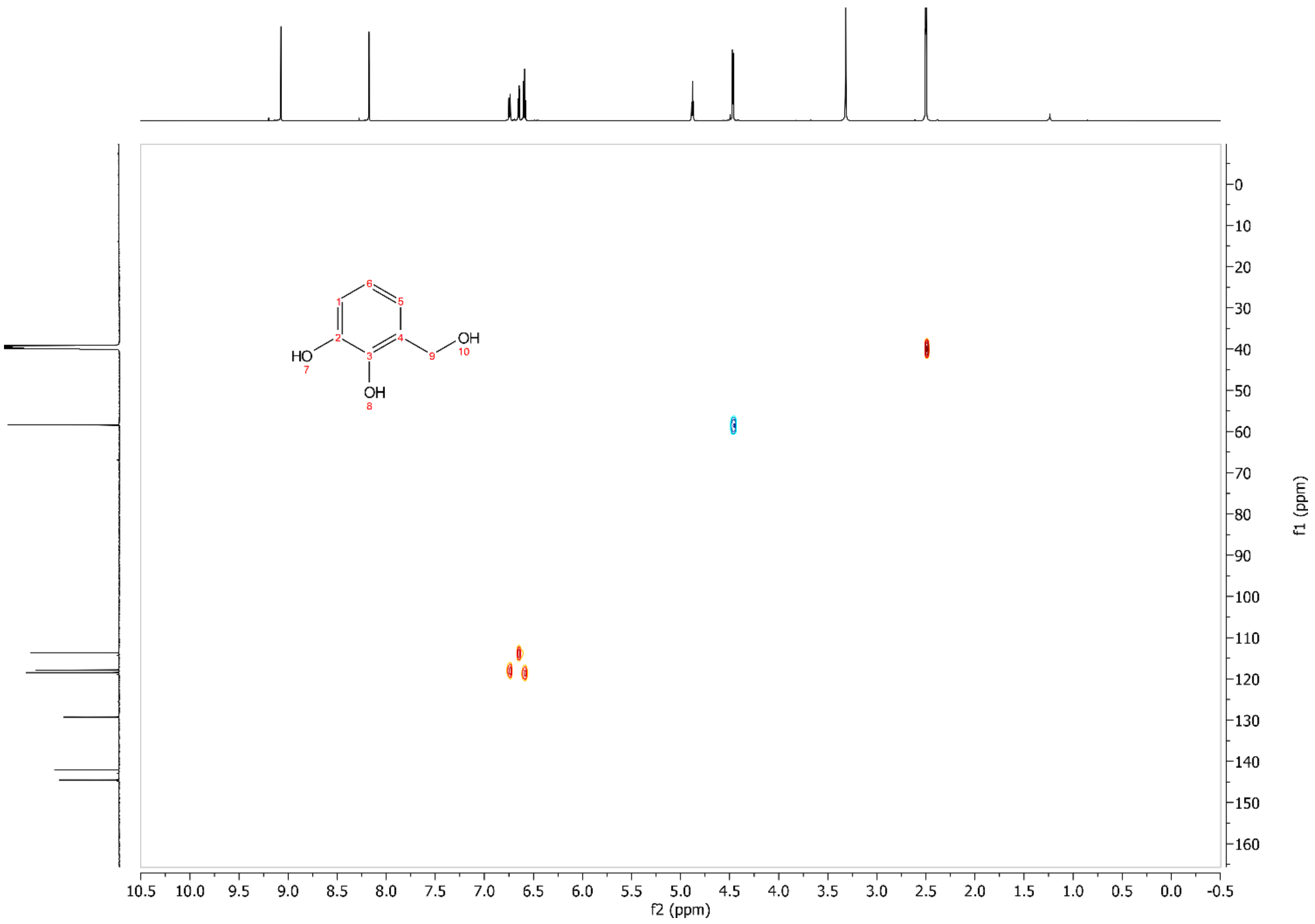

^{1}H-^{13}C HMBC of **15**

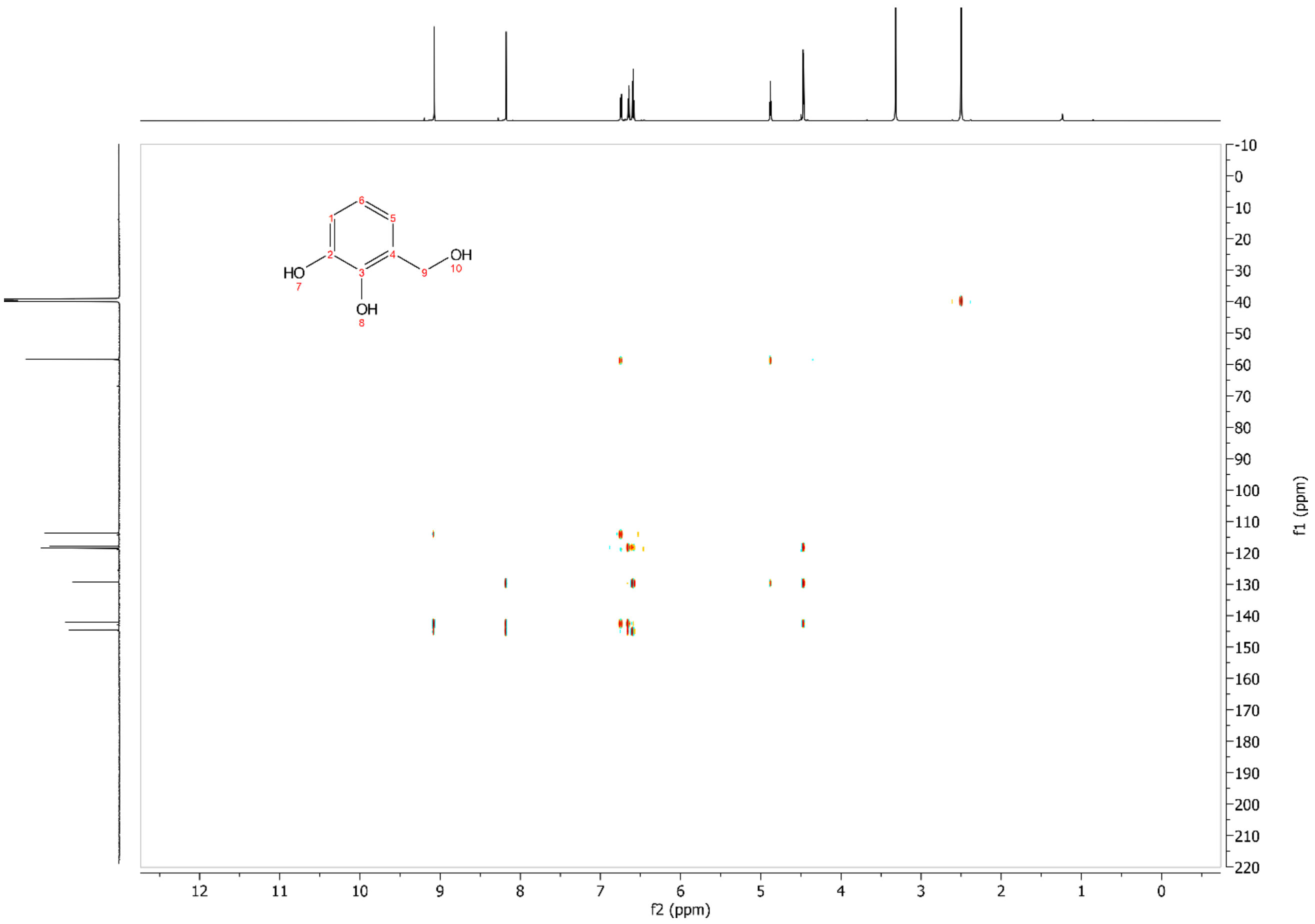

LC–MS spectra of **15**

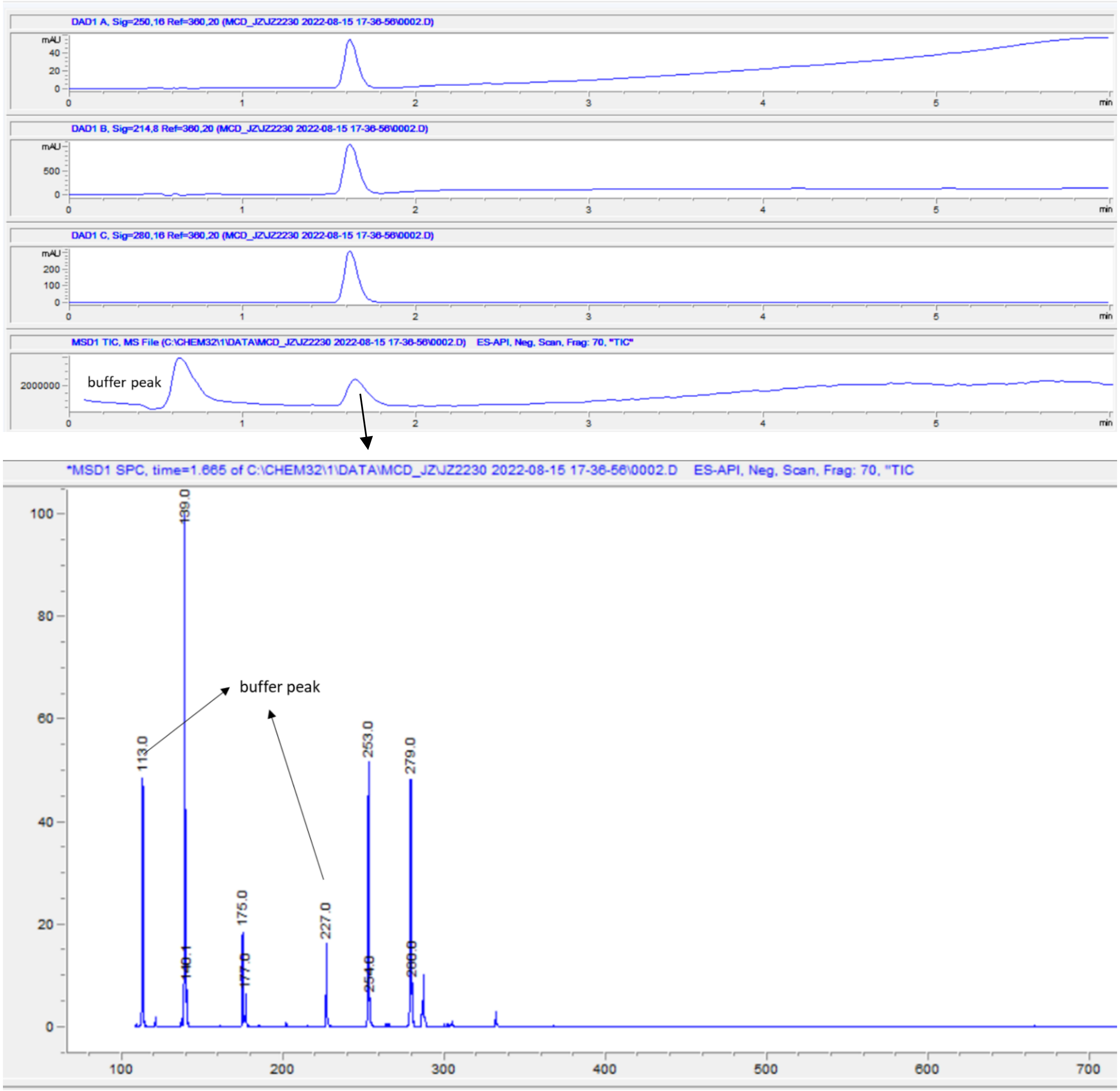

Protein sequence of p47phox^{SH3A-B}

The sequence of the human His-tagged p47phox$^{151-286}$ protein used in this study:

MHHHHHHHK LEVLFQGP DITGPIILQT YRAIANYEKT SGSEMALSTG DVVEVVEKSE SGWWFCQMKA KRGWIPASFL EPLDSPDETE DPEPNYAGEP YVAIKAYTAV EGDEVSLLEG EAVEVIHKLL DGWWVIRKDD VTGYFPSMYLQKSGQD

(https://www.uniprot.org/uniprotkb/P14598/entry)

Supplemental Figures

Supplemental Figure 1

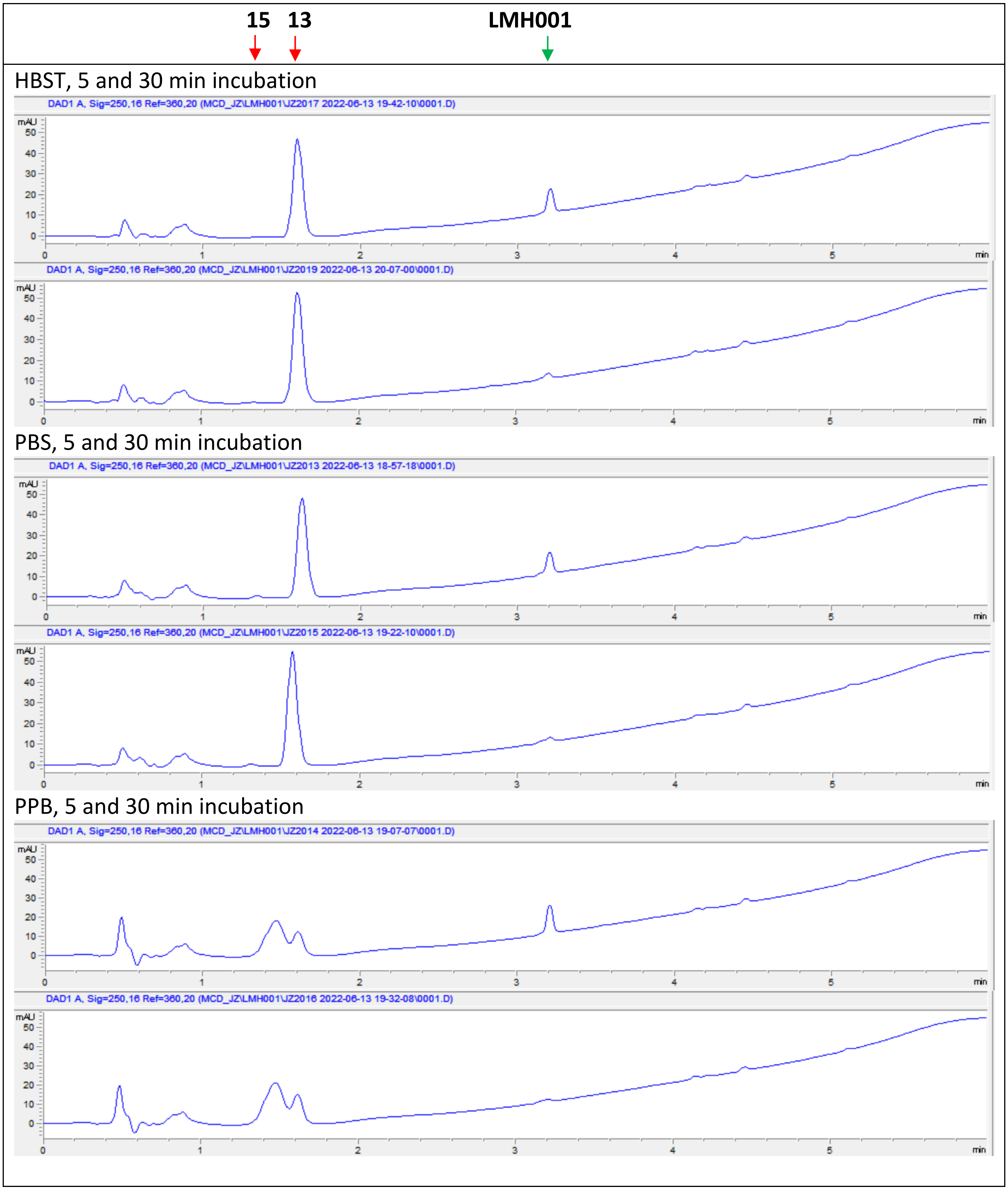

HBSS, 5 and 30 min incubation

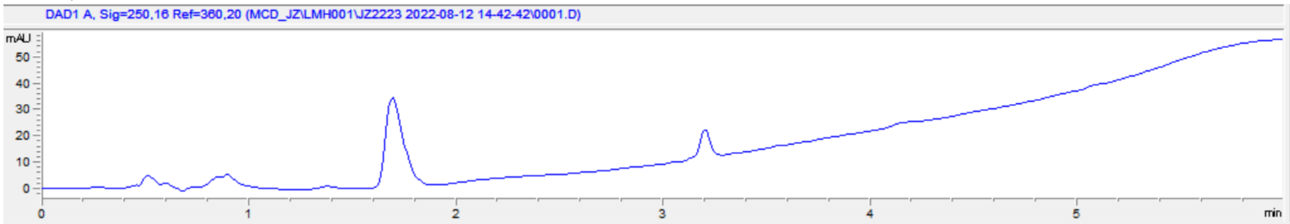

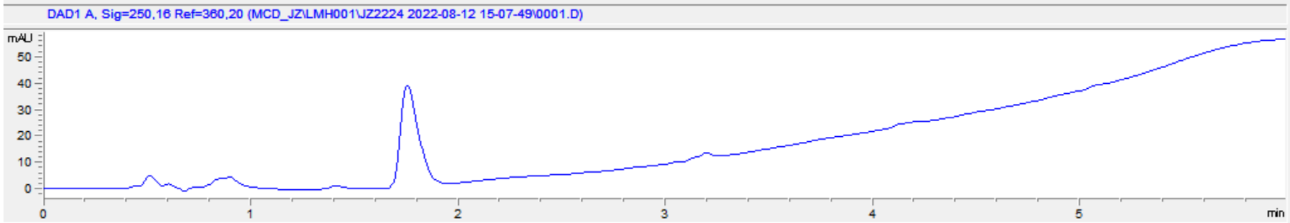

Hepes, 5 and 30 min incubation

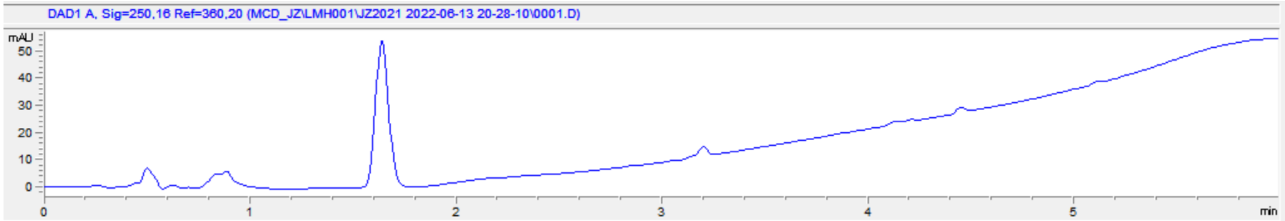

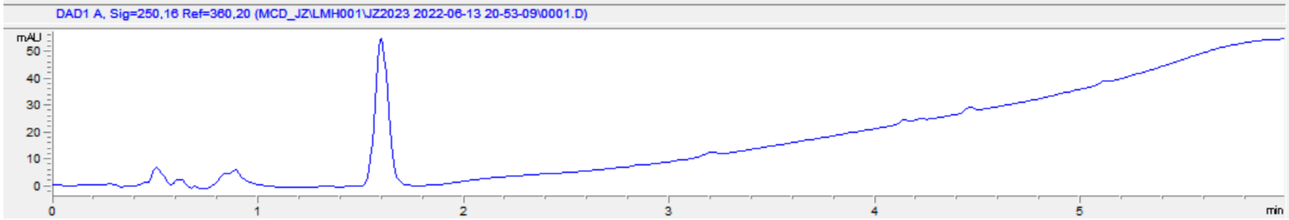

Tris, 5 and 30 min incubation

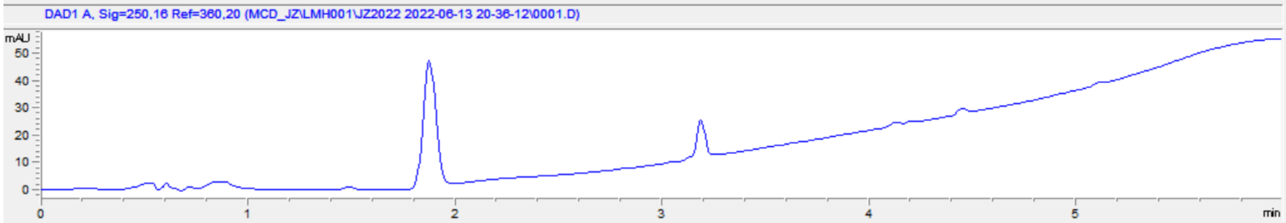

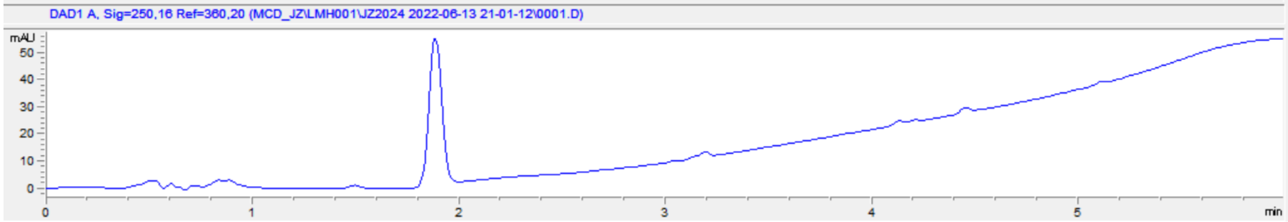

Water, 5 and 30 min incubation

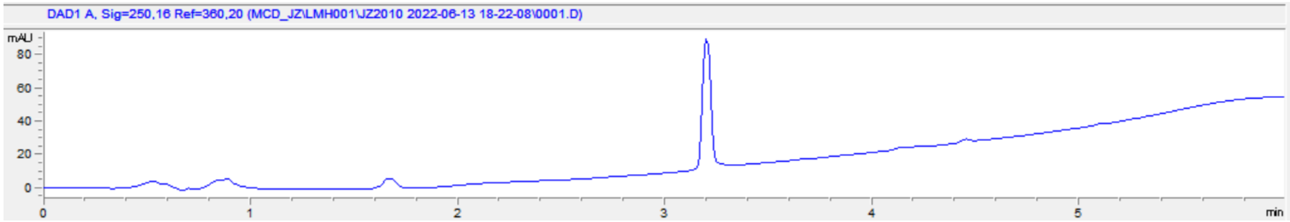

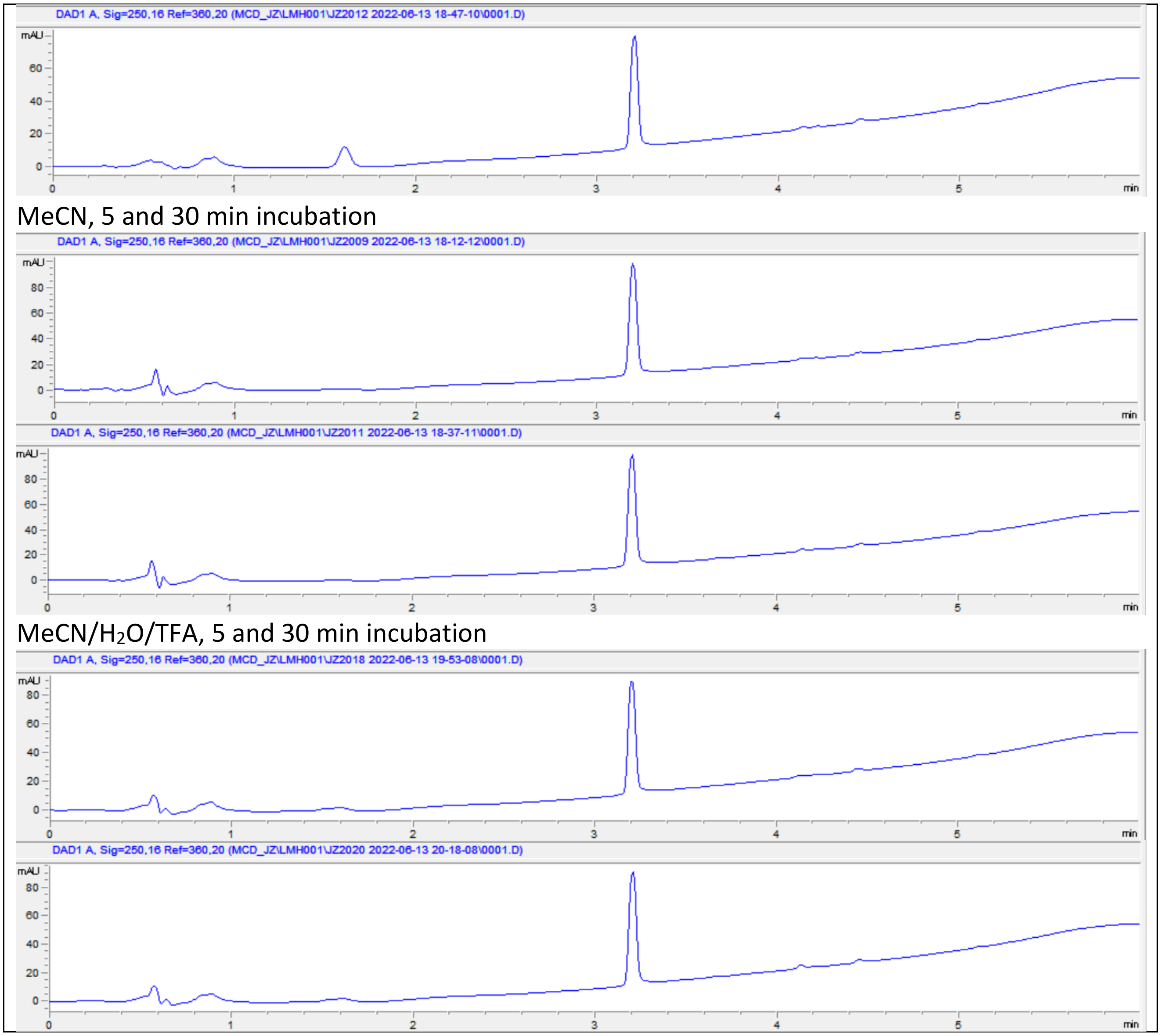

Supplemental Figure 1. LC–MS spectra of LMH001 in different buffers. Mass source parameters: Ion polarity, negative mode; drying gas flow, 12 L/min at 250 °C; nebulizer, 35 psig; VCap, 3000 V; Fragmentor, 70 V; mass scan range, 100–1000 m/z.

Supplemental Figure 2

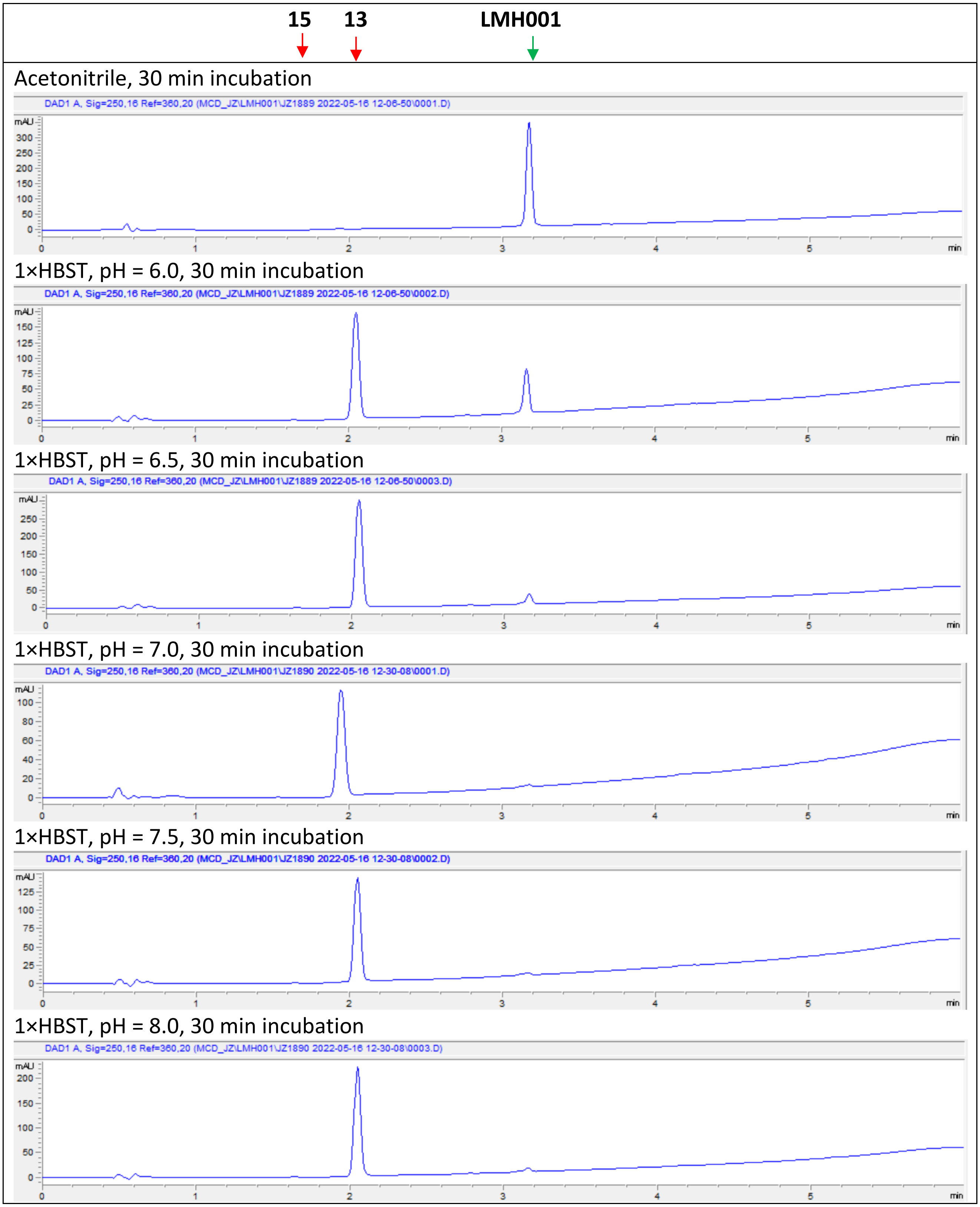

Supplemental Figure 2. LC–MS spectra of LMH001 in MeCN and 1×HBST with different pH values (6.0–8.0)

Supplemental Figure 3

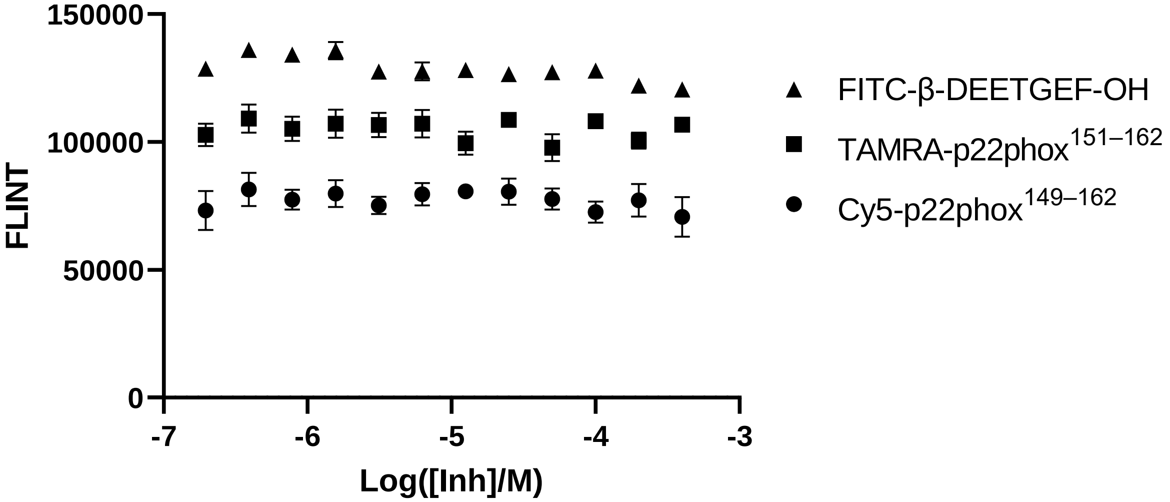

Supplemental Figure 3. The total fluorescence intensity (FLINT) of LMH001 incubated with Cy5-p22phox$^{149–162}$, TAMRA-p22phox$^{151–162}$ and FITC-β-DEETGEF-OH in 1×HBST in FP assay conditions to evaluate potential fluorescence interference of LMH001 with FP probes. FITC-β-DEETGEF-OH (β = beta-alanine) is a FITC labelled Nrf2 probe used to study another PPI - the Keap1/Nrf2 interaction (Tran et al., 2019).

Supplemental Figure 4

A

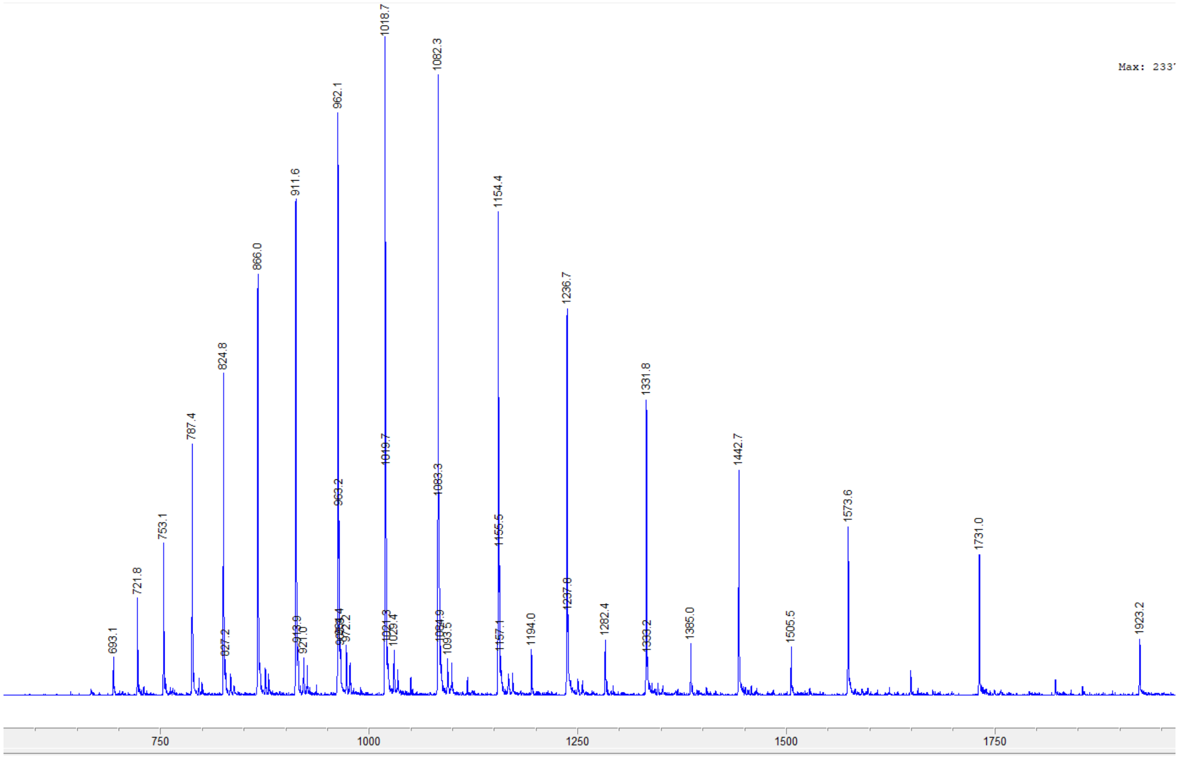

B

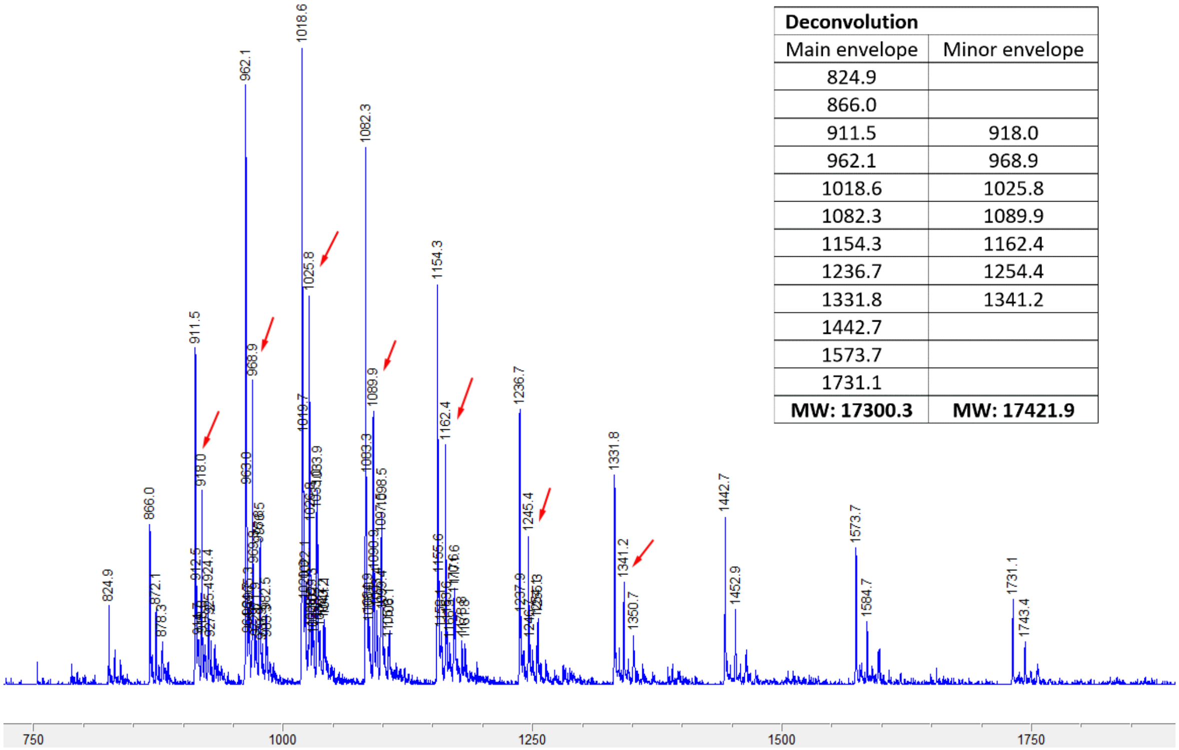

Deconvolution	
Main envelope	Minor envelope
824.9	
866.0	
911.5	918.0
962.1	968.9
1018.6	1025.8
1082.3	1089.9
1154.3	1162.4
1236.7	1254.4
1331.8	1341.2
1442.7	
1573.7	
1731.1	
**MW: 17300.3**	**MW: 17421.9**

C

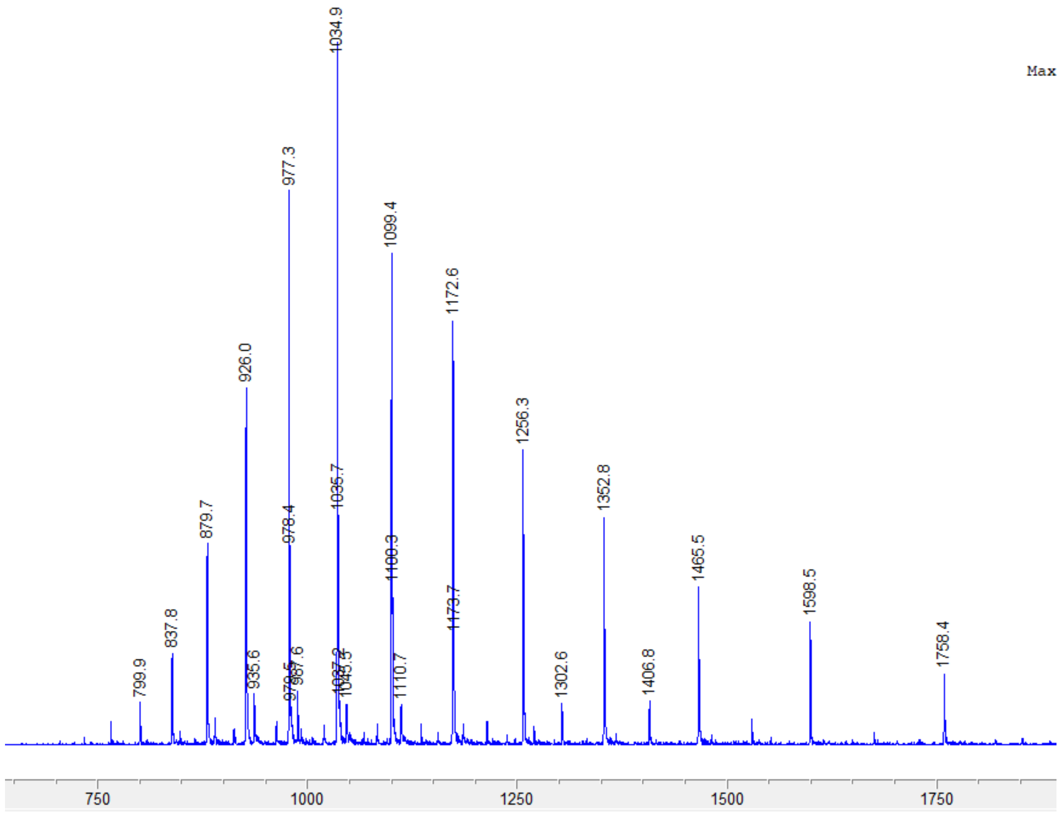
1034.9
Max
977.3
1099.4
1172.6
926.0
1256.3
1352.8
1035.7
978.4
879.7
1465.5
1598.5
837.8
1758.4
799.9
935.6
1110.7
1302.6
1406.8
750
1000
1250
1500
1750

D

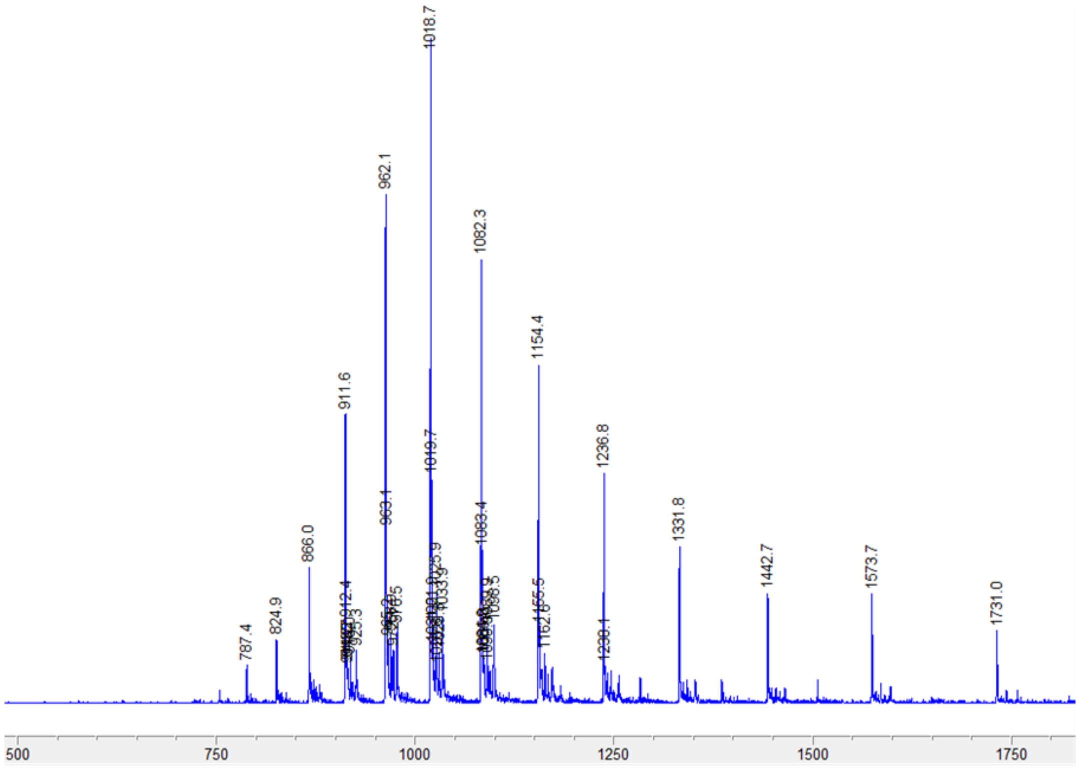
1018.7
962.1
1082.3
1154.4
911.6
1236.8
1331.8
866.0
1442.7
1573.7
824.9
1731.0
787.4
500
750
1000
1250
1500
1750

E

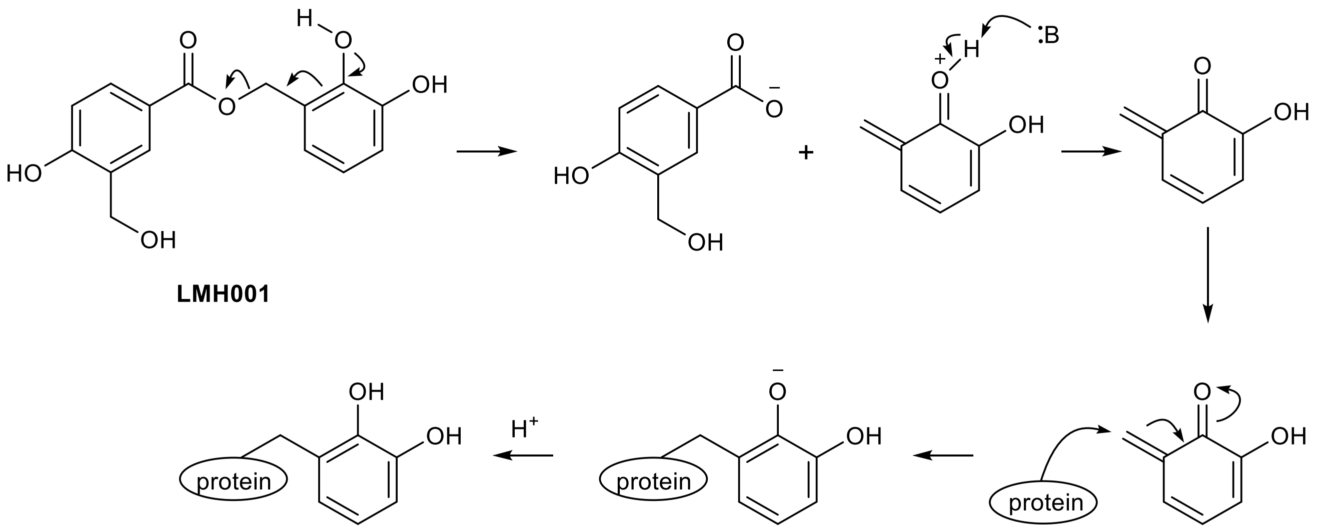

Supplemental Figure 4. Studies of covalent binding between p47phox$^{SH3A–B}$ and LMH001 or ebselen by LC–MS. Mass source parameters: Ion polarity, positive mode; drying gas flow, 12 L/min at 250 °C; nebulizer, 35 psig; VCap, 3000 V; Fragmentor, 70 V; mass scan range, 100–2000 m/z. Mass peak deconvolution was carried as described in literature (Winkler, 2010) (https://www.bioprocess.org/esiprot/esiprot_form.php). (A) LC–MS data of 0.2 mg/mL (11.6 µM) p47phox$^{SH3A–B}$ in HBST showed a MW of 17300.3 Da ± 0.15 Da (± SEM), corresponding to the theoretical MW of p47phox$^{SH3A–B}$ (17300.38 Da). (B) LC–MS data of 0.2 mg/mL p47phox$^{SH3A–B}$ with 200 µM LMH001 in HBST showed two mass envelopes, one is p47phox$^{SH3A–B}$ with MW of 17300.3 Da and another (see red arrows) is a protein adduct with MW of 17421.9 ± 0.29 Da (ΔMW relative to p47phox$^{SH3A–B}$ is 122 Da). (C) LC–MS data of 0.2 mg/mL p47phox$^{SH3A–B}$ with 200 µM ebselen in HBST showed a MW of 17574.0 Da ± 0.23 Da (ΔMW relative to p47phox$^{SH3A–B}$ is 274 Da corresponding to an ebselene-protein adduct). (D) LC–MS data of 0.2 mg/mL p47phox$^{SH3A–B}$ with 200 µM LMH001 in water showed a MW of 17300.3 Da corresponding the theoretical MW of p47phox$^{SH3A–B}$. (E) Proposed covalent binding mechanism of LMH001 with p47phox$^{SH3A–B}$. Similar to a phenolic Mannich base, the reaction involves an LMH001-derived quinone methide intermediate that reacts with nucleophiles from the protein leading to a +122 Da protein adduct.

Supplemental Figure 5

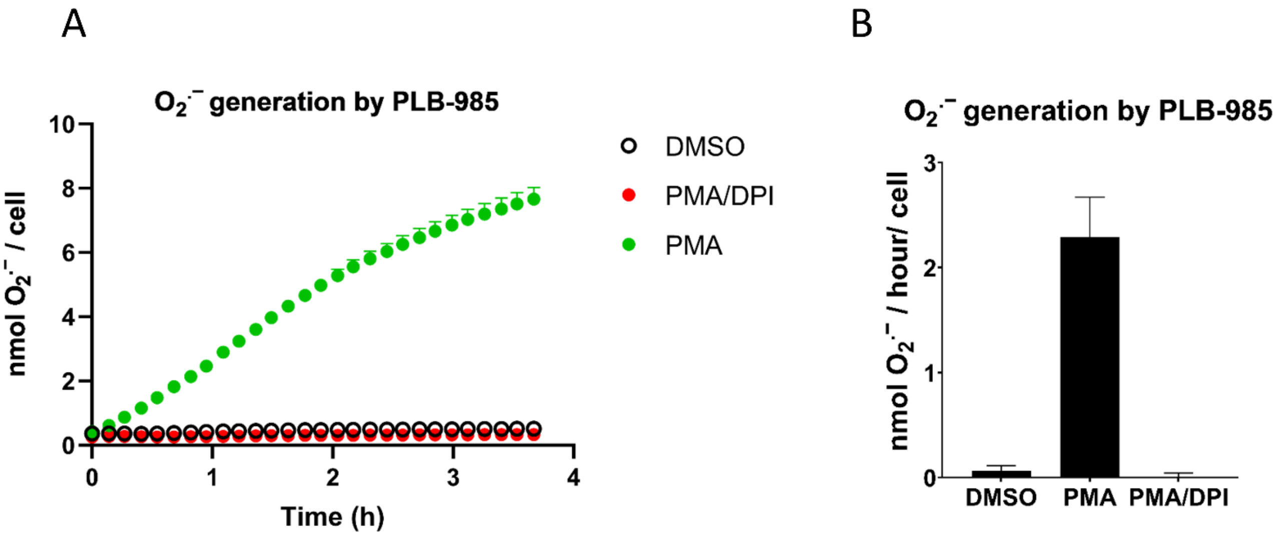

Supplemental Figure 5. (A) Traces of $O_2^{\bullet-}$ production following PMA activation of PLB-985 cells with and without DPI 10 µM. (B) Quantification of the $O_2^{\bullet-}$ production rate. Levels of $O_2^{\bullet-}$ were calculated based on the Beer-Lambert equation for WST-1: A = ε440 × l × C; where A: absorbance measured (AU); ε440 nm: 37000 M^{-1} cm^{-1}; l: 0.5 cm. Focal distance corresponds to 0.1 cm/10 µL for 384-w plate; C: $O_2^{\bullet-}$ concentration in molar.

References

Chang, M. Y. and Lin, C. Y. (2022). Bi(OTf)$_3$-Catalyzed Cyclocondensation of Oxygenated Arylacetic Acids with Salicyl Alcohols. Synthesis of 5-Oxadibenzo[*a*, *d*]cycloocten-6-ones. *Adv. Synth. Catal.*, 364, 3211–3224.

Leung, C. F. and Chow, H. F. (2017). Different Gelation and Self-Sorting Properties of Two Isomeric Polyamides Owing to the Parallel Versus Anti-Parallel Alignment of Backbone Dipoles. *Chemistry,* 23, 4827–4836.

Luu, Q. H., and Li, J. (2022). A C-to-O Atom-Swapping Reaction Sequence Enabled by Ni-Catalyzed Decarbonylation of Lactones. *Chem. Sci.,* 13, 1095–1100.

Stein, B. W., Cary, S. K., Berg, J. M., Birnbaum, E. R., Kozimor, S. A., Mocko, V., and Scott, B. L. (2018). A Series of F-Element Chelators; Diaza Crown Ethers Functionalized with Catecholate Binding Substituents. *J. Organomet. Chem.,* 857, 170–179.

Tran, K. T., Pallesen, J. S., Solbak, S. M. Ø., Narayanan, D., Baig, A., Zang, J., Aguayo-Orozco, A., Carmona, R. M. C., Garcia, A. D., and Bach, A. (2019). A Comparative Assessment Study of Known Small-Molecule Keap1-Nrf2 Protein-Protein Interaction Inhibitors: Chemical Synthesis, Binding Properties, and Cellular Activity. *J Med. Chem.,* 62, 8028–8052.

Winkler, R. (2010). ESIprot: A Universal Tool for Charge State Determination and Molecular Weight Calculation of Proteins from Electrospray Ionization Mass Spectrometry Data. *Rapid. Commun. Mass Spectrom.,* 24, 285–294.
